# Conjugated Inorganic–Organic Hybrid Polymers with p‐Block Elements

**DOI:** 10.1002/anie.202514344

**Published:** 2025-11-13

**Authors:** Anna‐Lena Thömmes, David Scheschkewitz

**Affiliations:** ^1^ Krupp‐Chair for General and Inorganic Chemistry Saarland University 66123 Saarbrücken Germany

**Keywords:** Heavier main group elements, Inorganic p‐block elements, Inorganic–organic hybrid polymers, Multiple bonds, Organic electronics

## Abstract

Conjugated polymers with small band gaps and the ensuing semiconductivity are increasingly used as active layers in organic electronics. In order to achieve purpose‐built polymers with specific band gaps and optimized charge transport properties, covalent doping with inorganic main group heteroelements has proven to be a promising strategy during the past 20 years. This review highlights recent advances in the introduction of single heteroatoms of Group 13 to 15 into the organic conjugated main chain of such hybrid polymers, with a particular focus on the fine‐tuning of the photophysical properties for applications in electronic devices. The emerging field of polymers containing p‐block multiple bonds is covered in the second part, aiming to provide a comprehensive overview of research in the arena of conjugated polymers incorporating main group motifs in the main chain. The electronic and structural properties are significantly altered by the interaction at the inorganic‐organic interface and can be optimized using regio‐ or stereoisomers and targeted post‐functionalization. A general trend toward the heavier elements is evident, pushing the door wide open to a vast uncharted terrain with huge potential for further developments in inorganic‐organic hybrid polymers for electronics and sensing materials with advanced properties.

## Graphical abstract

The influence of various p‐block element motifs on the photophysical properties of conjugated inorganic‐organic hybrid polymers is surveyed regarding applications in organic electronics. Recent advances in the use of heavier elements as well as heavier multiple bonds and the development of diverse (post‐)functionalization strategies suggest ample scope for exciting future developments.

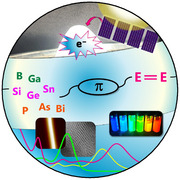

## 1 Introduction

Polymeric materials are characterized by their unmatched versatility, as manifest in the variety of applications for almost every aspect of our lives and in the continuously increasing yearly production.^[^ ^1^, ^2^, ^3^, ^4^, ^5^, ^6^, ^7^ ^]^ Conjugated derivatives become more and more important as tailorable materials for (opto‐)electronic devices.^[^ ^8^, ^9^, ^10^ ^]^ The groundbreaking discovery of semiconductivity in such polymers was acknowledged with the Nobel Prize in Chemistry in 2000 to A. J. Heeger, A. G. MacDiarmid, and H. Shirakawa “for the discovery and development of conductive polymers”.^[^ ^11^ ^]^ The advantageous mechanical properties in combination with semiconductivity and high charge carrier mobilities of some organic materials even outperform conventional semiconductors based on, for instance, bulk silicon or germanium.^[^ ^12^ ^]^ Prominent examples of polymer electronics include organic light emitting diodes (OLEDs),^[^ ^13^, ^14^, ^15^, ^16^ ^]^ organic solar cells (OSCs),^[^ ^17^, ^18^, ^19^, ^20^ ^]^ organic field effect transistors (OFETs),^[^ ^21^, ^22^, ^23^ ^]^ and capacitors,^[^ ^24^ ^]^ as well as medical and pharmaceutical devices, such as artificial muscles and bio/chemosensors.^[^ ^25^, ^26^, ^27^, ^28^, ^29^, ^30^, ^31^, ^32^ ^]^ Apart from light weight and (often) flexibility, low‐cost production and low toxicity, polymers offer crucial advantages, such as facile processability and disposal as well as possibilities for modular combination of building blocks. A main focus of current research on polymer electronics is to increase the device efficiencies by enhancing charge transport and exciton formation in the emissive layer of OLEDs.^[^ ^33^, ^34^, ^35^, ^36^ ^]^ State‐of‐the‐art multilayer polymer electronics, however, face considerable challenges such as the need of multiple deposition steps potentially compromising the structure of underlying layers, and the presence of heterojunctions resulting in decreased charge transport and efficiency and hence the requirement of high operating voltages.^[^ ^33^, ^37^ ^]^ In recent years, single‐component devices were investigated as an innovative strategy to address these issues.^[^ ^38^, ^39^, ^40^ ^]^

To allow for the combination of the desired wide range of properties in a single material, the search for new derivatives is a must. The ongoing expansion of the library of accessible structural motifs thus increasingly grants access to purpose‐built materials with specific energy band levels and adjustable charge generation and transport properties. Notably, modern digital devices—still often based on conventional purely inorganic semiconductors—comprise almost the entirety of the periodic table.^[^ ^41^ ^]^

In recent years, covalent doping of organic polymers with “inorganic” elements of the p‐block has come to the fore. Targeted electronic and geometric modifications through the incorporation of Group 13 or heavier Group 14 and 15 motifs into the main chain offer a variety of possibilities for adjusting the photophysical properties and charge carrier mobilities. This has been partially outlined in previous reviews, which, however, touch upon the broad scope of recent advances in conjugated inorganic‐organic hybrid polymers only peripherally.^[^ ^42^, ^43^, ^44^, ^45^, ^46^, ^47^, ^48^, ^49^ ^]^

**Figure 1:**
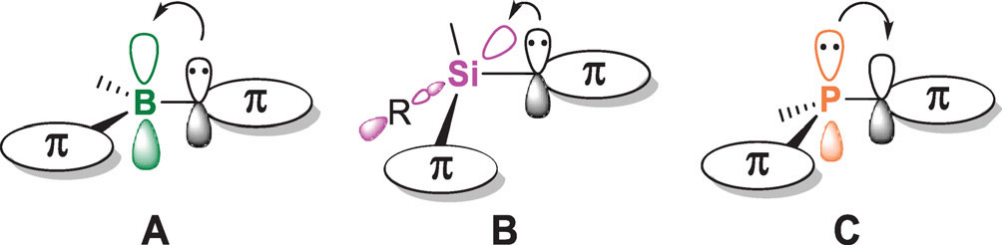
Schematic representation of different types of interaction of Group 13 to 15 elements with the organic π‐conjugated scaffold.

Band gap tuning through covalent doping of the polymer main chain is either achieved *directly* through incorporation of the heteroelement as part of the conjugation path or *indirectly* by favorable interaction of the π‐system with lone pairs or vacant orbitals of the heteroelements in the backbone. In both cases, various conjugation mechanisms have been realized as a consequence of the different electronic structures of the applied p‐block motifs (Figure 1), with implications for the photophysical properties, the reactivities, and the adjustability through post‐functionalization. In Group 13, the empty p‐orbital of the sp^2^‐hybridized heteroatom center is crucial for the interaction with the extended π‐system, allowing for p,π‐conjugation (**A**) between the “inorganic” and the “organic” part. In Group 14, the typically low‐lying σ*‐orbitals of sp^3^‐hybridized moieties act as electron acceptors, resulting in σ,π‐conjugation (**B**). Pnictogen motifs are characterized by their lone pairs of electrons constituting electron‐donating groups for n,π‐conjugation (**C**).

This review aims to provide a comprehensive overview of conjugated, semiconducting inorganic–organic hybrid polymers with functional p‐block motifs as potential part of the conjugation path. We focus on such structures with embedded single heteroatoms or diheteroatomic multiple bonds. Particular attention is paid to the effect of the doping motif on the optoelectronic properties. Conversely, synthetic procedures are only discussed in detail for unconventional approaches beyond the well‐established cross‐coupling techniques.

In view of the rapid developments involving different areas of p‐block chemistry, it is no surprise that subfields at different stages of maturity have been reviewed before, particularly in the case of boron, as the lightest element in Group 13, numerous conjugated polymers with monoboron centers have been reported.^[^ ^50^, ^51^, ^52^, ^53^, ^54^, ^55^, ^56^, ^57^, ^58^, ^59^, ^60^, ^61^, ^62^, ^63^, ^64^, ^65^, ^66^, ^67^, ^68^, ^69^, ^70^ ^]^ Selected recent developments concerning boron polymers are nonetheless included here, and a comprehensive overview of the less investigated gallium derivatives and the scattered reports on aluminum‐containing polymers is provided. Derivatives with coordinatively saturated boryl side‐chains^[^ ^71^, ^72^, ^73^, ^74^, ^75^, ^76^ ^]^ are, however, not considered due to the absence of suitable vacant or lone‐pair orbitals at the Group 13 element for interaction with the conjugation path. The heavier Group 14 elements are also on the rise,^[^ ^77^, ^78^ ^]^ beginning to complement the vast variety of the well‐known lighter poly(silane)s.^[^ ^42^, ^79^, ^80^, ^81^, ^82^, ^83^, ^84^, ^85^, ^86^ ^]^ While poly(silole)s, as the most prominent examples, are hence only briefly introduced with references to corresponding reviews,^[^ ^81^, ^82^, ^84^, ^85^, ^87^, ^88^, ^89^, ^90^, ^91^, ^92^, ^93^, ^94^, ^95^ ^]^ a broad overview of the different types of reported germanium‐based derivatives is given. Details on recent developments on the far less investigated poly(stannane)s are provided, and diatomically bridged congeners are introduced. Recent developments in poly(pnictane)s^[^ ^42^, ^96^, ^97^, ^98^, ^99^, ^100^, ^101^ ^]^ and their post‐functionalization are discussed, including poly(arsane)s, which have drawn attention more recently.^[^ ^102^ ^]^

Finally, conjugated polymers with multiply bonded heteroatom motifs in the main chain are by far less common in comparison and are discussed in the second part of this review as an emerging field. Particularly, in the case of homo‐ and heteronuclear Group 14 and 15 multiple bonds, there are only very few examples, despite the development of a wide range of functionalized unsaturated molecular species featuring such motifs in unperturbed form.^[^ ^103^, ^104^, ^105^, ^106^, ^107^, ^108^, ^109^, ^110^, ^111^, ^112^, ^113^, ^114^, ^115^, ^116^, ^117^, ^118^, ^119^ ^]^ A notable exception to this scarcity is poly(iminoborane)s containing zwitterionic B═N bonds with partial double bond character. Recent studies suggest the rather counterintuitive involvement of the BN unit in the conjugation path.

With the virtually unlimited possibilities of main group chemistry at hand, an extraordinarily rich tool‐box is available for further improvement of the corresponding hybrid polymer electronics. This review intends to highlight these possibilities and encourage the further development of the methodology necessary for a modular combination of these various motifs.

## 2 Single Heteroatoms Embedded in the Main Chain

### 2.1 Group 13: Poly(gallane)s & Recent Developments of Poly(borane)s

The overwhelming majority of studies with Group 13 elements employ boron as an electron‐deficient center,^[^ ^50^, ^51^, ^52^, ^53^, ^54^, ^55^, ^56^, ^57^, ^58^, ^59^, ^60^, ^61^, ^62^, ^63^, ^64^, ^65^, ^66^, ^67^, ^68^, ^69^, ^70^ ^]^ which is presumably due to the ease of synthesis and a priori enhanced air and moisture stability of the resulting compounds compared to the heavier congeners. In Chujo's seminal work on derivatives of boron‐doped poly(phenylenevinylene)s, the p,π‐conjugation across the vacant p‐orbitals at the boron centers was demonstrated for the first time by bathochromic shifts of the polymers’ UV/vis absorptions in comparison to a corresponding monomer.^[^ ^120^, ^121^ ^]^ The use of different organic linking units (phenylene, biphenylene, fluorene, furan, thiophene, and pyridine) provokes variable red‐shifts up to orange‐red fluorescence in the case of pyridine (*λ_abs_* = 350–450 nm, *λ_em_* = 420–590 nm). More recently, considerable bathochromic shifts of the absorption and luminescence (*λ_abs_* = 440–460 nm, *λ_em_* = 500–570 nm) were achieved for oligoacetylene‐bridged poly(borane)s with aromatic side chains instead of aromatic linkers in the main chain.^[^ ^122^ ^]^ The incorporation of a 9,10‐diboraanthracenyl unit in such a polyacetylene chain resulted in similar absorption and emission properties (*λ_abs_* = 410 nm, *λ_em_* = 518 nm).^[^ ^123^ ^]^

**Scheme 1:**
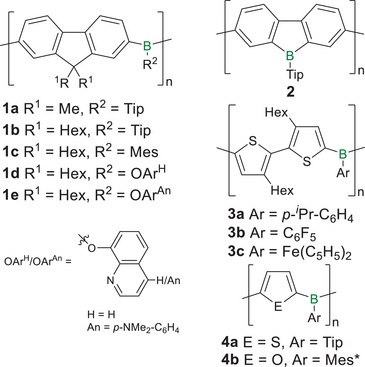
Examples of poly(borane)s with variable linking units: fluorene (**1a–e**), bithiophene (**3a–c**), thiophene (**4a**), furan (**4b**) and poly(borafluorene) **2** (Tip = 2,4,6‐triisopropylphenyl, Mes = 2,4,6‐trimethylphenyl, Mes* = 2,4,6‐tri‐*tert*‐butylphenyl).

Highly emissive fluorene‐bridged poly(borane)s **1a‐c** (Scheme 1) and a related donor‐acceptor copolymer (*λ_abs_* = 380–420 nm, *λ_em_* = 390–440 nm, quantum yields: 75%–95%) were investigated for their coordination behavior toward nucleophiles, which resulted in blue‐shifted absorption (Δ*λ* ∼ 80 nm) and quenching of the blue‐violet fluorescence.^[^ ^124^, ^125^, ^126^ ^]^ Notably, in the case of **1d,e** with donor substituents, only weak blue‐shifted green and red fluorescence is observed (*λ_abs_* = 394, 444 nm, *λ_em_* = 504, 639 nm, quantum yields: 21%, 0.1%). The emission in poly(borafluorene) **2** and a corresponding vinylene copolymer is far more red‐shifted (*λ_abs_* = 474, 495 nm,* λ_em_* = 586, 612 nm) and hence switchable fluorescence in the visible range (between yellow and blue) was observed upon base coordination.^[^ ^127^ ^]^

Jäkle reported the thiophene‐bridged analogues **3a‐c** (Scheme 1) obtained by Sn/B exchange reaction of distannyl bithiophenes and dibromo arylboranes.^[^ ^128^ ^]^ The absorption and, in the case of **3a,b**, fluorescence (*λ_abs_* = 390–410 nm, *λ_em_* = 491, 529 nm) lie in the range spanned by Chujo's polyacetylene derivatives. Coordination of pyridine to the Lewis acidic boron centers results in fluorescence quenching and a strongly blue‐shifted absorption (*λ_abs_* ∼ 300 nm). An alternative synthetic strategy was recently implemented by Helten et al.: Si/B exchange reactions of disilyl thiophenes and dibromo boranes in the presence of catalytic amounts of Me_3_SiNTf_2_ (Tf = SO_2_CF_3_) provide poly(borane)s **4a,b** with thiophene or furan linker units.^[^ ^129^, ^130^ ^]^

Derivatives with homologous thienyl or furyl chains of variable lengths as linking units between the borane centers show increasing bathochromic shifts with increasing spacer lengths (S: *λ_abs_* = 470–490 nm, *λ_em_* = 450–590 nm; O: *λ_abs_* = 360–430 nm, *λ_em_* = 400–490 nm) and a systematic red‐shift for the polythiophenes in comparison.^[^ ^131^, ^132^ ^]^ In thin films of the polythiophenes, even larger bathochromic shifts were observed (*λ_em_* = 500–660 nm). Considerably high quantum yields were achieved, in particular for the polyfurans (S: 12%–38%, O: 32%–87%). A trend toward lower fluorescence intensity was observed with increasing Group 16 element content. For the bithiophene‐ and bifuran‐bridged polymers, the quantum yields were highest, presumably due to increased rigidity in combination with still relatively low S and O amounts. Donor–acceptor polymers with dithienylborane acceptor units containing the electron‐withdrawing ^F^Mes substituent at the boron were employed in OSCs, resulting in reasonable power conversion efficiencies of up to 2.83%.^[^ ^133^ ^]^

**Figure 2:**
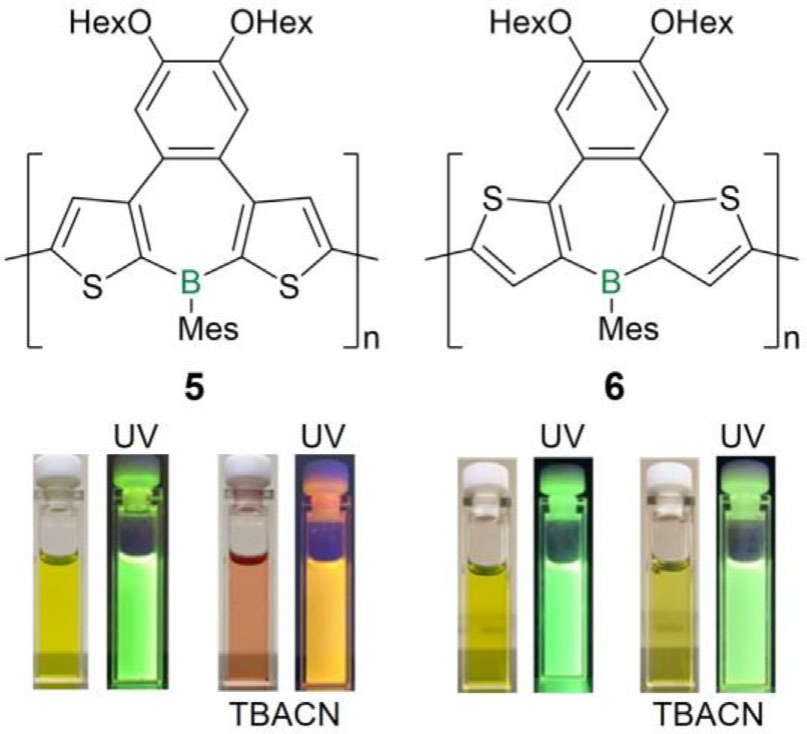
Top: Regioisomeric poly(dithienoborepin)s **5** and **6**. Bottom: Solutions of **5** and **6** under ambient light and UV light (Mes = 2,4,6‐trimethylphenyl), respectively, before and after addition of 2 equiv of tetrabutylammonium cyanide (TBACN). Adapted with permission from Ref. [134]. Copyright 2021 Royal Society of Chemistry.

Recently, Ohshita et al. investigated the differences in the electronic structures, the resulting optical properties and the effect of Lewis base coordination in poly(dithienoborepin)s **5** and **6** with fused dihexoxybenzenes in the backbone (Figure 2).^[^ ^134^ ^]^ The two regioisomers **5** and **6** were obtained by Stille cross‐coupling of the corresponding distannyl‐ and dihalo‐substituted monomers. The Hückel aromatic borepin scaffold with sterically protecting and electron‐rich fused thiophene rings shows air and water stability.^[^ ^135^ ^]^ Both poly(borepin)s exhibit conjugation along the main chain, manifest in significant bathochromic shifts of the absorption (**5**:* λ_abs_* = 475 nm, **6**: *λ_abs_* ∼ 450 nm) and fluorescence (**5**: *λ_em_* = 525, **6**: *λ_em_* = 500 nm) compared to the monomers (*λ_abs_* = 355–395 nm, *λ_em_* = 405–415 nm). Thin films of **5** and **6** evoke additional red‐shifts by Δ*λ* = 10–90 nm, presumably due to intermolecular interactions in the solid state.

As expected and previously described for structurally related thiophene fused borepin polymers,^[^ ^135^, ^136^ ^]^ the conjugation path in polymer **6** with the sulfur atoms in β‐position to boron extends along the backbone rather than the boron centers. The frontier orbitals show major contributions of the benzene backbone and, in the LUMO, no discernible contribution is found at the boron centers, in stark contrast to **5** where the sulfur atoms reside in α‐position to boron. Unsurprisingly, the cross‐conjugated position of boron in **6** exerts a smaller influence than the fully π‐conjugated position in **5**. As a consequence, cyanide coordination at the boron centers leads to significant changes in the optical properties of **5** (Figure 2), while the spectra of **6** are considerably less affected. For both, **5** and **6**, hypsochromically shifted (Δ*λ* ∼ 100 nm) absorption bands appear after cyanide addition, yet in the case of **5**, their intensity is significantly weakened and an additional broad bathochromically shifted band with an onset at *λ_abs_* ∼ 650 nm was observed, presumably due to charge transfer (CT) transitions from the tetracoordinate boron centers. Accordingly, the fluorescence of cyanide‐coordinated **5** is also bathochromically shifted from green to orange (Δ*λ* = 130 nm, Figure 2), whereas the emission maximum of **6** is not influenced. The weaker donor pyridine induces similar effects. UV/vis absorption and emission vanish completely upon cyanide addition to the corresponding monomers, due to interruption of the conjugation and the lack of similar CT transitions.

Pu and Ren et al. reported on the coordination of pyridine derivatives to the boron centers in poly(trithienylborane) networks in which the boron atoms act as bridges between the 2‐positions of thiophene rings and are thus incorporated in the conjugation path.^[^ ^137^ ^]^ In these cases, the donor coordination provokes a hypsochromic shift of the emission (by Δ*λ* = 60 nm from *λ_em_* = 660 nm) through inhibition of the conjugation across the previously vacant p‐orbitals at boron. A cross‐linked derivative was applied as a photocatalyst for hydrogen production.

These studies demonstrate the potential of heteroelement‐based conjugated polymers for the (in some cases reversible) fine‐tuning of the band gap: small changes in the chemical environment of the heteroelement, such as the coordination of a nucleophile, exert considerable influence on the optical properties of the materials. The responsiveness of the materials toward such changes can be controlled, for example, by employing different regioisomers.

**Figure 3:**
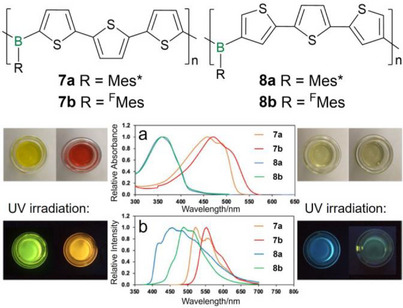
Top: Regioisomeric poly(thienylborane)s **7a,b** and **8a,b** (Mes* = 2,4,6‐tri‐*tert*‐butylphenyl, ^F^Mes = 2,4,6‐tris(trifluoromethyl)phenyl). Bottom: Solutions of **7a,b** and **8a,b** under ambient light and UV light, and corresponding representative absorption (a) and fluorescence spectra (b). Adapted with permission from Ref. [138]. Copyright 2023 Wiley VCH.

Similarly, bora‐substitution next to the thiophene rings in 2‐position in poly(thienylborane)s **7a,b** (Figure 3) results in more effective conjugation between the π‐system and the empty p‐orbitals at the boron centers in comparison to substitution in 3‐position in the corresponding regioisomers **8a,b**.^[^ ^138^ ^]^ The energy of the LUMO levels and thus the gap to the mostly unaffected highest occupied molecular orbital (HOMO) are substantially decreased in **7a,b**, resulting in huge bathochromic shifts of the absorption maxima by Δ*λ* = 100–125 nm (*λ_abs_* = 460–485 nm) compared to those of **8a,b** (*λ_abs_* = 360). Although not explicitly discussed by the authors, the somewhat smaller shifts of the fluorescence maxima by Δ*λ* = 25 to 70 nm and accordingly smaller Stokes shifts in the case of **7a,b** plausibly result from structural relaxation of the excited states: the aryl substituents at the boron centers (R in Figure 3), which are perpendicular to the thiophene rings in the electronic ground state according to the single crystal X‐ray structures of corresponding monomers, could approach a more favorable conformation closer to coplanarity in **8a,b**, in line with the slightly broader emission bands compared to those of **7a,b** (Figure 3). The very bulky Mes* and ^F^Mes substituents were employed to provide considerable air and moisture stability and even resistance against oxidants, acids, and bases.^[^ ^139^ ^]^ Formal substitution of the *tert*‐butyl groups of the supermesityl substituent Mes* (**7a**,**8a**) by CF_3_ groups (**7b**,**8b**) allows for a more delicate fine‐tuning, providing smaller red‐shifts of the absorption and emission in the range of Δ*λ* = 2–50 nm.

**Scheme 2:**
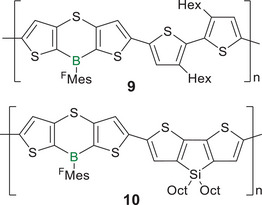
Alternating dithienoborane copolymers **9** and **10** with bisthiophene and dithienosilole units, respectively, and sulfur‐bridges connecting the thiophene rings in the borane units (^F^Mes = 2,4,6‐tris(trifluoromethyl)phenyl).

Adachi and Ohshita et al. employed a sulfur‐bridge to enforce coplanarity of the thiophene rings,^[^ ^140^ ^]^ a generally favorable feature to enable efficient charge transport in semiconducting thin‐film materials.^[^ ^141^ ^]^ In compounds **9** and **10** (Scheme 2), the introduction of the aromatic thiaborin ring results in the stabilization of the HOMO and a hypsochromic shift of the absorption by Δ*λ* = 30–40 nm in comparison to unbridged derivatives.^[^ ^131^, ^142^ ^]^ Nonetheless, considerable conjugation is deduced from the presence of huge bathochromic shifts of Δ*λ* = 50–180 nm compared to the monomer and model compounds. The silole moiety induces a red‐shift by Δ*λ* = 90 nm from **9** to **10** by further increasing the system's planarity and thereby the conjugation cross section. In addition, the enhanced planarity also induces π‐stacking in thin films of the resulting polymers **9** and **10** (Scheme 1) due to π–π and S–S interactions. The aggregate formation was suggested to explain the appearance of additional red‐shifted absorption bands and shoulders between *λ_abs_* = 500 and 600 nm in the solution and thin film UV/vis spectra, respectively. Accordingly, these absorption bands are absent in solutions of **9** that had been filtered through 0.2 µm membranes. Monomers with considerably shorter π‐systems showed no indication of intermolecular stacking. While the polymers **9** and **10** exhibit intense green fluorescence in solution (*λ_em_* = 532, 563 nm) and red fluorescence in thin films (*λ_em_* = 669, 686 nm), the monomers only exhibit weak fluorescence at room temperature but blue‐green phosphorescence at 77 K. Very recently, the same group reported room temperature phosphorescence in solution and in the solid state through introduction of BiPh_2_ end groups in the conjugation path of thiophene‐substituted boranes to enhance intersystem crossing.^[^ ^143^ ^]^

In contrast to poly(borane)s, conjugated hybrid polymers of the heavier Group 13 elements are scarce. The only known aluminum‐based examples so far contain porphyrin or salen ligands employing N─Al─N and O─Al─O moieties, respectively, in the otherwise organic π‐conjugated main chain.^[^ ^144^, ^145^, ^146^ ^]^ The resulting microporous structures allow for CO_2_ capture (∼8 wt%)^[^ ^144^ ^]^ and applications as efficient catalysts in the conversion of CO_2_ to carbonates. Salen‐coordinated gallium oligothiophene copolymers were obtained previously through electropolymerization of the corresponding monomers with thiophene end groups.^[^ ^147^ ^]^ The O─Ga─O containing polymer with four‐coordinate cationic gallium centers served as a precursor for Ga_2_S_3_ nanoparticles, in contrast to the corresponding neutral five‐coordinate derivative. The metal centers, however, do not contribute to the conjugation in these systems and only act as Lewis acidic reaction sites,^[^ ^148^, ^149^ ^]^ as is also known for related boron‐based polymers.^[^ ^150^, ^151^ ^]^

**Figure 4:**
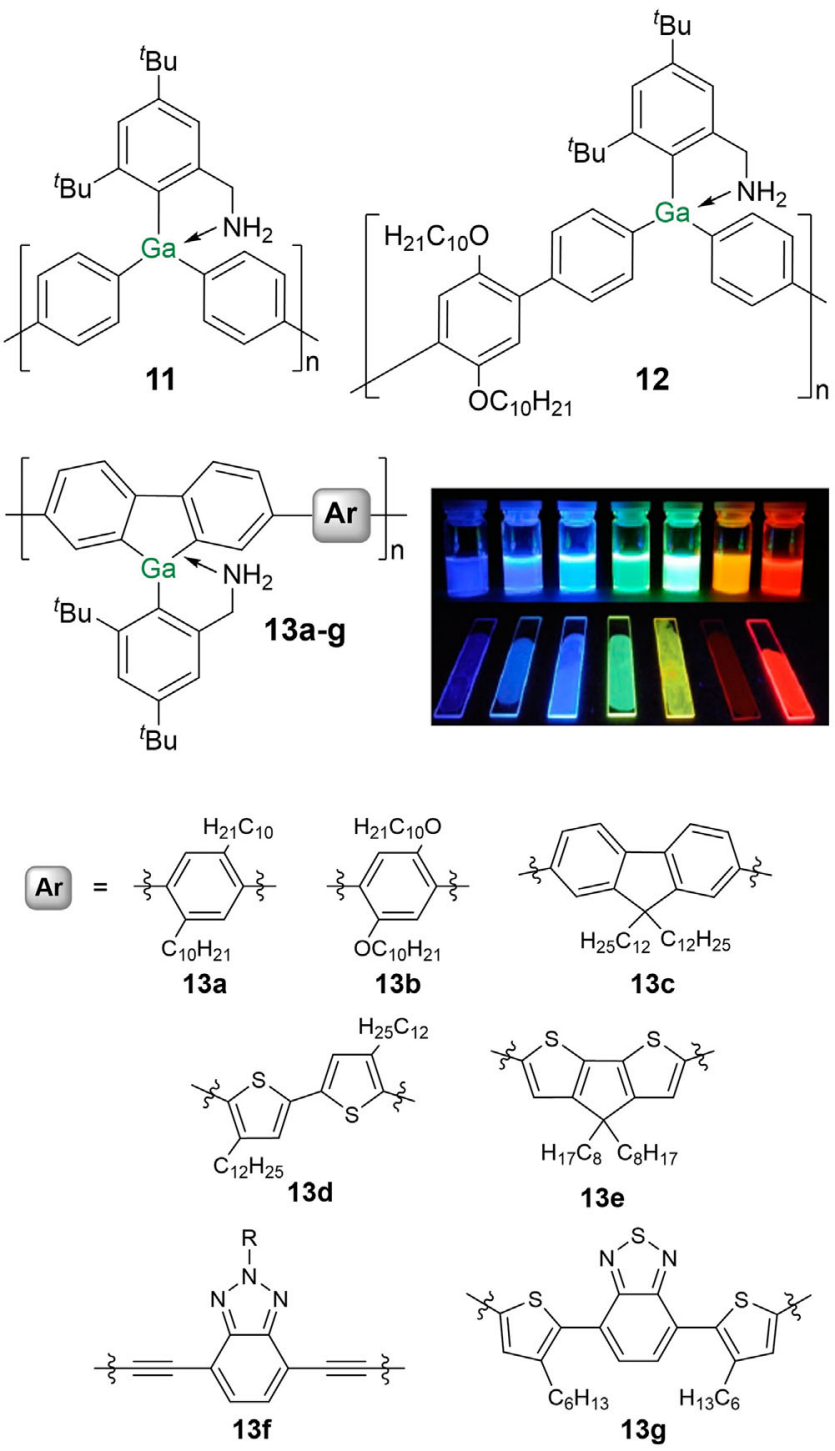
Arylene‐bridged poly(gallane)s **11** and **12** and poly(gallafluorene)s **13a–g** (R = 2‐decyl‐tetradecane). Solutions and thin films of polymers **13a–g** under UV irradiation. Adapted with permission from Ref. [152]. Copyright 2015 American Chemical Society.

Poly(gallane)s **11** and **12** are the only polymers with arylene‐bridged gallane units in the conjugation path (Figure 4).^[^ ^153^ ^]^ They are obtained by transition metal catalyzed C─C coupling of the corresponding bifunctionalized monomers. A comparison of the UV absorption of **11** and **12** (*λ_abs_* = 275, 325 nm) to that of the corresponding monomer (*λ_abs_* = 242 nm), of a monomeric model compound with a biphenyl substituent at gallium (*λ_abs_* = 263 nm) and of the dimer (*λ_abs_* = 269 nm) revealed a systematic bathochromic shift. DFT calculations on a molecular model compound confirmed the delocalization of the lowest unoccupied molecular orbital (LUMO) across the gallane center. Both, **11** and **12**, show emission in the UV range (**11**: *λ_em_* = 378 nm, **12**: *λ_em_* = 392 nm).

The only known examples of gallium incorporation into a fluorene scaffold are the gallafluorene copolymers **13a–g**.^[^ ^152^ ^]^ Depending on the employed electron‐deficient comonomer units, variable red‐shifts of the UV/vis absorption and fluorescence are provoked (*λ_abs_* = 330–480 nm, *λ_em_* = 380–640 nm). The most red‐shifted absorptions were obtained for cyclopentadithiophene (**13e**), ethynylene‐substituted benzotriazole (**13f**), and benzothiadiazole (**13g**) linking units. As a result, the fluorescence of **13a–g** covers the whole range of the visible spectrum (Figure 4). In the case of **13e,f**, additional red‐shifted bands assigned to intermolecular transitions were observed in thin films (and in a concentrated solution of **13f**). Possibly, this effect is due to the enhanced planarity through the extended π‐bridges and intermolecular interactions established through additional S‐ and N‐centered lone‐pairs of electrons.

A trend toward the heavier elements is evident from these scarce Al‐ and Ga‐containing examples. First developments concern the catenation of In and Tl,^[^ ^154^, ^155^ ^]^ however, no corresponding conjugated polymers are known as of yet.

### 2.2 Group 14: Rise of the Heavier Elements in Hybrid Poly(tetrelane)s

Simple poly(silane)s exhibit σ‐conjugation due to low‐lying σ*‐orbitals at the tetracoordinate silicon atoms. The resulting semiconductivity, first discovered by West et al. for poly((phenylmethyl)silane) in the presence of SbF_5_ as strong electron acceptor,^[^ ^156^ ^]^ leads to UV light absorption and emission.^[^ ^81^, ^157^, ^158^ ^]^ These materials have been known for decades and have been applied in various fields, for instance, as photoresist and adhesion‐supporting materials in coatings and microlenses, as sensors for the detection of explosives, as radical initiators or as hydrogen storage materials and, most importantly, in conducting or semiconducting devices, such as OLEDs or OSCs.^[^ ^159^, ^160^, ^161^, ^162^ ^]^ The semiconducting properties are, however, dependent on the conformation of the Si─Si bonds, which influences the conjugation along the polymer chain^[^ ^157^, ^158^, ^163^ ^]^—in notable contrast to mostly planar π‐conjugated materials.

**Figure 5:**
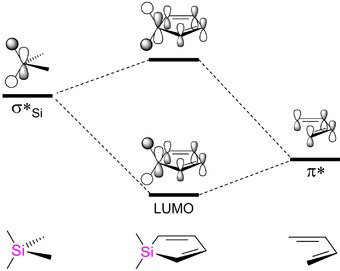
Qualitative orbital correlation diagram for 1,1‐dimethylsilole based on PM3 calculations by Tamao et al.^[^ ^87^ ^]^

Combinations of σ‐ and π‐conjugation were explored in anticipation of potential fine‐tuning of the band gaps by simple changes in the repeat units’ structures. In heavier analogues of pyrroles and related compounds, such as fluorenes, the heteroelement influences the HOMO‐LUMO gap *indirectly* through σ*‐π* mixing of low‐lying σ*‐orbitals (Figure 5). The associated decrease of the LUMO levels and, to a lesser extent, the HOMO energies results in smaller band gaps.^[^ ^42^, ^87^, ^164^, ^165^ ^]^ For instance, the formal substitution of the CH_2_ group in cyclopentadiene for a SiH_2_ group was calculated to lower the LUMO level by 1.29 eV, but the HOMO by only 0.44 eV.^[^ ^87^ ^]^

Poly(silole)s or derivatives thereof containing phenylene, acetylene, or thiophene spacers are the most prominent representatives and have shown superior performance in electronic devices compared to purely organic congeners.^[^ ^82^, ^84^, ^85^, ^87^, ^88^, ^89^, ^90^ ^]^ Numerous examples have been isolated and, in some cases, applied to OLEDs, OSCs, and OFETs due to their high charge carrier mobilities.^[^ ^91^, ^92^, ^93^ ^]^ Poly(silole)s typically show blue fluorescence, and their rigid propeller‐like structure prevents quenching of emission and allows for aggregation‐induced fluorescence, predestining them for applications as bio/chemosensors.^[^ ^81^, ^94^, ^95^ ^]^

**Scheme 3:**
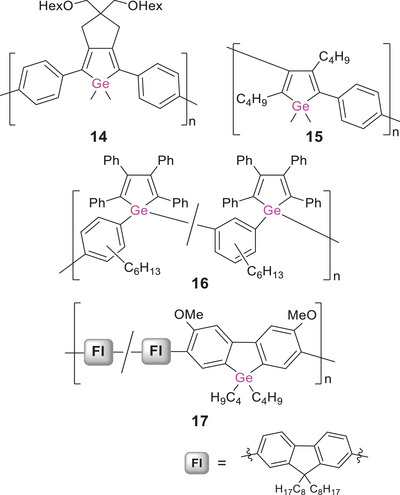
Representative examples of poly(phenylene germole)s **14**–**16** bridged via different positions and a poly(germafluorene) **17**.

Due to the d‐block contraction, germanium analogues often exhibit even superior electronic properties and a better chemical stability than silicon congeners.^[^ ^90^, ^166^, ^167^, ^168^, ^169^ ^]^ There are different types of poly(germole)s according to the ring positions of the atoms connecting the monomer units. In a seminal work by Tilley et al., the first derivatives—ring‐bridged poly(2,5‐diphenylgermole) **14** (Scheme 3) and related oligomers—were reported to exhibit green fluorescence (*λ_em_* = 490–500 nm) and absorption in the visible range (*λ_abs_* = 400 to 440 nm).^[^ ^170^ ^]^ The considerably lower LUMO energy induced by the presence of germanium in the backbone results in remarkable bathochromic shifts with increasing chain length (by up to Δ*λ* = 80 nm for the absorption), in particular compared to the corresponding monomeric model compounds, which show blue fluorescence (*λ_em_* = 450–460 nm) and absorb in the UV (*λ_abs_* = 360–380 nm).

Polymer **14** is prepared by nickel‐mediated C─C coupling of dihalides of the 2,5‐diphenylgermole monomer, in turn obtained through metallacycle transfer of the corresponding zirconacyclopentadiene with GeCl_4_. This kind of transmetalation of metalacyclic structures has been applied for the construction of various poly(heterole)s in the following years (vide infra). Conveniently, the transmetalation is mostly implemented as post‐functionalization, as, for instance, in the synthesis of poly(2,4‐diphenylgermole) **15**, for which a titanacyclopentadiene polymer was transmetalated with GeCl_4_.^[^ ^171^ ^]^ Random copolymer **16** constitutes an exceptional example in which the repeat units are connected via the germanium atoms.^[^ ^172^ ^]^ It was obtained by polycyclotrimerization of the diacetylene‐substituted germole with 1‐octyne. In contrast to typical poly(heterole)s, the *direct* incorporation of the germanium center into the conjugation path results in a strongly red‐shifted absorption band in the visible range at *λ_abs_* ∼ 540 nm. While this was taken as a hint toward extended conjugation via σ,π‐interactions between the arylene bridges and the germole rings across the germanium centers, the red‐shifted band likely originates from aggregation‐induced intermolecular transitions. Further evidence for the formation of aggregates was provided by the increased emission intensity in thf solutions at lower temperatures (*λ_em_* = 490 nm, *λ_exc_* = 410 nm).

The incorporation of germanium into a poly(fluorene) was achieved in 2009 via Suzuki coupling of a bis(pinacolboryl)fluorene and a dibrominated germafluorene.^[^ ^173^ ^]^ The obtained random germafluorene‐fluorene copolymer **17** (Scheme 3) exhibits intense blue fluorescence and absorption in the UV range (*λ_em_* = 418, 441 nm, *λ_abs_* = 388 nm), as does the corresponding homopolymer (*λ_em_* = 415 nm, *λ_abs_* = 380 nm).^[^ ^174^ ^]^ While the optical band gaps are relatively large (2.9, 3.0 eV), donor–acceptor copolymers with thiophene‐bridged benzothiadiazole or diketopyrrolopyrrole acceptor units reduce them to 1.6–1.8 eV and thus confer reasonable hole mobilities (up to 8·10^−3^ cm^2^ V^−1^ S^−1^) and power conversion values (up to 2.8%) in OFETs and OSCs. The incorporation of thiophene units has proven advantageous in poly(dithienogermole)s. These offer a variety of possibilities for fine‐tuning the band gap, the charge carrier mobilities and the photophysical properties through modification of the substitution pattern at the heteroatom or the thiophene rings and employment of different copolymers.^[^ ^89^, ^90^, ^166^, ^167^, ^175^, ^176^, ^177^, ^178^, ^179^, ^180^, ^181^, ^182^, ^183^ ^]^

**Scheme 4:**
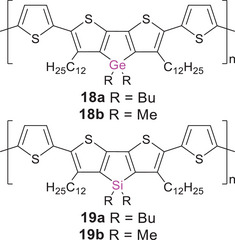
Poly(dithienogermole)s **18a,b** and poly(dithienosilole)s **19a,b** with thiole spacer units.

In a recent study, the influence of the Group 14 atoms in poly(dithienotetrole)s **18a** and **19a** (Scheme 4) on intersystem crossing (ISC) rates while maintaining similar optical absorption properties, oxidation potentials and charge carrier mobilities was elucidated.^[^ ^184^ ^]^ The germole derivative exhibits a smaller ISC rate, presumably due to enhanced coplanarity of the rings, resulting in higher exciton yields in donor‐acceptor blends with a fullerene derivative.

**Scheme 5:**
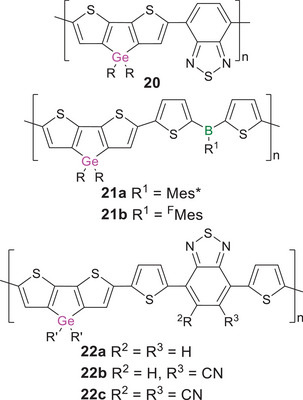
Poly(dithienogermole)s **20**–**22** with different linking units (R = 2‐ethylhexyl, R′ = 2‐octyldodecyl, Mes* = 2,4,6‐tri‐*tert*‐butylphenyl, ^F^Mes = 2,4,6‐tris(trifluoromethyl)phenyl).

The increased bond length of the Ge─C bond and therefore reduced steric strain were assumed to account for this structural effect in an earlier work by Kim and Heeney et al. on the related donor–acceptor copolymer **20** with a benzothiadiazole linking unit instead of the thiophene units in **18** and **19** (Scheme 5).^[^ ^167^ ^]^ Bathochromic shifts of the thin film absorption maxima compared to the corresponding silole derivative were observed, in addition to an increased degree of crystallinity and a higher charge carrier mobility in an OSC device. For both, **20** and the corresponding silole, the longest wavelength absorptions (Ge: *λ_abs_* = 773 nm, Si: *λ_abs_* = 754 nm) lie in the far red of the visible spectrum and were assigned to charge‐transfer transitions between the heterole donor part and the benzothiadiazole acceptor unit as well as to intermolecular transitions due to aggregation. End‐capping with phenyl groups was shown to result in favorable additional intermolecular interactions and with that to enhanced ordering in thin films and thus increased charge carrier mobilities.^[^ ^180^ ^]^

Further fine‐tuning of the photophysical properties can be achieved through modification of the substitution pattern at the heteroatom, as was shown for **18a,b** and **19a,b**.^[^ ^185^ ^]^ In fact, methyl substitution results in a substantial bathochromic shift of the absorption (Ge: *λ_abs_* = 548 nm, Si: *λ_abs_* = 559 nm) and the red thin‐film emission (Ge: *λ_em_* = 706 nm, Si: *λ_em_* = 701 nm) by Δ*λ* = 40 to 60 nm compared to the corresponding butyl‐substituted derivatives **18a** and **19a** (Ge:* λ_abs_* = 509 nm, *λ_em_* = 649 nm, Si:* λ_abs_* = 508 nm,* λ_em_* = 652 nm), likely due to more effective intermolecular π‐stacking in the dimethyl poly(tetrole)s **18b** and **19b**.

Employing dithienylborane units instead of benzothiadiazole units (as in **20**) as the electron‐deficient moieties in donor–acceptor‐type polymers **21a,b** provided access to the more blue‐shifted range of the spectrum with a hypsochromic shift of the absorption from the far red to *λ_abs_* = 510–540 nm: the polymers appear red instead of blue and show red fluorescence (*λ_em_* = 600–650 nm).^[^ ^142^ ^]^ Thiophene‐bridged benzothiadiazole/dithienogermole copolymers **22a–c** have been applied in OSC devices by Heeney et al. A comparison reveals that slight changes in the backbone substitution pattern of the acceptor unit can have considerable effects: the monocyano‐substituted derivative **22b** leads to a power conversion efficiency of 6.5%, twice as high as with unsubstituted **22a**.^[^ ^182^ ^]^ Disubstitution in **22c**, however, hampers the exciton generation significantly by further lowering the LUMO to a level at which electron transfer to the additionally employed fullerene acceptor is prevented, resulting in an efficiency of <1%. In line with a narrowing of the band gap, the thin film absorption is bathochromically shifted with an increasing number of cyano substituents (**22a**: *λ_abs_* = 676 nm, **22b**: *λ_abs_* = 786 nm, **22c**: *λ_abs_* = 833 nm).

**Scheme 6:**
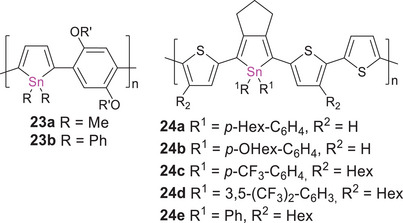
The only known examples of regioregular poly(stannole)s: **23a,b** with phenylene and **24a‐e** with thiophene linking units (R′ = 2‐ethylhexyl).

The corresponding tin analogues have been far less investigated in comparison to their lighter congeners. In analogy to the synthesis of poly(germole)s, the first poly(stannole)s, bridged with phenylene or biphenylene linkers in the 2,4‐ and 2,5‐positions of the stannole ring, were obtained by transmetalation of the corresponding titanacyclopentadiene polymer with SnCl_4_.^[^ ^186^ ^]^ The LUMO energy levels are lowered by the tin center in a comparable manner as in an analogous poly(germole), resulting in absorption maxima in the UV range between *λ_abs_* = 281 and 289 nm, which tail into the visible region (∼400 nm) and correspond to similarly large band gaps of ∼3 eV. Regioregular derivatives **23a,b** are connected exclusively via the 2,5‐positions of the stannole ring (Scheme 6).^[^ ^187^ ^]^ They were prepared in corresponding post‐functionalizations of regioregular titanacyclopentadienes and give rise to substantially smaller band gaps of ∼ 2 eV (*λ_abs_* = 457, 491 nm, **23a**: *λ_em_* = 542 nm).

Remarkable bathochromic shifts by up to Δ*λ* = 130 nm compared to molecular model compounds were observed in these cases as well as in the only other examples of known poly(stannole)s **24a–e** with thiophene linkers, corresponding to small band gaps (down to 1.6 eV in thin films).^[^ ^188^, ^189^ ^]^ The air‐ and moisture‐stable poly(thienylstannole)s were prepared by Stille cross‐coupling of diiodothienylstannoles (with different substituents at the central tin atom) with the corresponding distannylthiophenes. The absorption of the *para*‐hexylphenyl and *para*‐hexyloxyphenyl‐substituted derivatives **24a,b** (*λ_abs_* = 556, 560 nm) is bathochromically shifted in comparison to **24c,d** (*λ_abs_* = 532, 522 nm), despite much shorter chain lengths (**24a,b**: *X_n_* = 6.3; **24c,d**: *X_n_* = 10–12) and although electron‐withdrawing groups at the tin center typically reduce the HOMO–LUMO gap. Notably, **24a‐d** display weak red fluorescence with similar shifts of the emission maxima (**24a,b**: *λ_em_* = 716, 717 nm, **24c,d**: *λ_em_* = 654, 655 nm). It was assumed that the additional *n*‐hexyl groups in the backbone of **24c,d** reduce the planarity of the system and hence diminish effective conjugation. In derivative **24e**, the solubility‐enhancing *n*‐hexyl groups are located at the thiophene rings as in **24c,d**, but **24e** exhibits less electron‐withdrawing phenyl substituents at tin and shorter chain lengths (**24e**: *X_n_* = 9, **24c,d**: *X_n_* = 10–12).^[^ ^189^ ^]^ The absorption maximum of **24e** (*λ_abs_* = 536 nm) lies, however, in between those of **24a,b** and **24c,d**, possibly due to electronic and steric effects of the CF_3_ groups: the electron‐withdrawing influence could diminish conjugation along the polymer chain through increased interaction of the π‐electrons with the aryl substituents at tin. The steric demand of the CF_3_ groups might additionally reduce the co‐planarity of the system.

The implementation of Si or Ge into the π‐conjugation path of poly(amide)s, poly(ester)s, poly(imide)s, poly(azomethine)s and related compounds confers improved solubility in organic solvents as compared to the organic congeners while maintaining good thermal stability and appropriate glass transition temperatures, hence rendering them superior materials for thin‐film applications.^[^ ^190^, ^191^, ^192^, ^193^, ^194^, ^195^, ^196^, ^197^, ^198^, ^199^, ^200^, ^201^, ^202^ ^]^ While their syntheses and thermal properties have been extensively investigated during the past decades, the (opto‐)electronic properties, crucial for applications in electronic devices, moved into focus more recently. Germanium‐doped oligo(urethane)s show blue fluorescence due to the low‐lying σ*‐orbitals at the tetrelane moieties and resulting σ,π‐conjugation along the polymer chain (as discussed above for poly(silole)s), but exhibit relatively large band gaps of ∼ 3.8 eV (*λ_abs_* = 270–310 nm, *λ_em_* = 350–360 nm).^[^ ^203^ ^]^ Although poly(azomethine)s with silylene bridges exhibit similarly large band gaps of 3.1–3.5 eV,^[^ ^204^ ^]^ these poly(germane)s become conductive in the form of thin films. Phenyl groups at the heteroatom or phenylene instead of thiophene bridges give rise to band gaps as small as 2.0 eV,^[^ ^205^, ^206^ ^]^ resembling those of poly(thiophene)s used in optoelectronics.^[^ ^207^, ^208^ ^]^

**Scheme 7:**
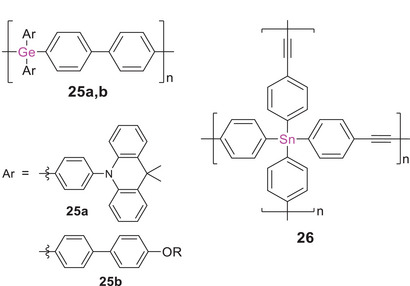
Representative examples of σ,π‐conjugated polymers with germanium atoms embedded in the conjugation path of linear π‐conjugated organic polymers **25a,b**, and a corresponding poly(stannane) **26** (R = 2‐ethylhexyl).

The σ,π‐conjugated poly(biphenylgermane) **25a** (Scheme 7) was just recently employed as a host material in a blue light emitting OLED with an excellent external quantum efficiency of 24%.^[^ ^209^ ^]^ In combination with an organic co‐host and variable guest compounds, blue, red, green, and white emitting devices were obtained.^[^ ^210^ ^]^ In a comparative study of related poly(biphenyltetrelanes) containing different Group 14 heteroelements (C, Si, Ge, and Sn), the germanium‐based analogue **25b** exhibited the best device performance, despite smaller hole and electron mobilities than the silicon congener.^[^ ^211^ ^]^

Only recently, poly(stannane) **26** with ethynylene and arylene linking units was obtained by Sonogashira cross‐coupling of tetra(ethynylphenyl)stannane and diiodobenzene in a 1:2 ratio.^[^ ^212^ ^]^ Depending on the reaction conditions, i.e., base and catalyst employed, catalyst load, solvent, temperature, and reaction time, **26** was obtained as microporous or nonporous insoluble material with Brunauer–Emmet–Teller surface areas between 2 and 750 m^2^ g^−1^. The largest pore sizes (1.6–2.8 nm) were achieved with NEt_3_ as base and high catalyst load (2 mol% Pd(PPh_3_)_2_Cl_2_) in toluene. The corresponding anisotropically shaped particles were about 500 nm in size according to scanning electron microscopy. Fluorescence maxima (in the solid state and in suspension) are located at *λ_em_* = 500–540 nm or at *λ_em_* = 460–480 nm. The preference for either range presumably depends on the macroscopic structure. Although no conclusive trend was discernible, the applied base as well as the catalyst load seem to have a considerable impact on the emission. In comparison to the intense blue fluorescence (solution: *λ_em_* = 407 nm, solid state: *λ_em_* = 452 nm) of *para*‐phenylethynylbenzene with triphenylstannyl end groups as a molecular model compound, the emission of the polymers is bathochromically shifted, independent of the reaction conditions.

Although the increasing interest in hybrid polymers with germanium and tin confirms an apparent trend toward the heavier elements, reports on analogues of lead, the heaviest stable Group 14 element, are still lacking. The incorporation of covalently bonded σ‐conjugated Group 14 moieties into π‐conjugated (polymeric) frameworks continue to be extensively investigated:^[^ ^79^, ^213^, ^214^, ^215^, ^216^, ^217^, ^218^, ^219^, ^220^, ^221^ ^]^ various representatives with alkenyl, alkynyl and aryl bridging units^[^ ^222^, ^223^, ^224^, ^225^, ^226^, ^227^, ^228^, ^229^, ^230^, ^231^, ^232^, ^233^ ^]^ as well as furane and thiophene rings were reported.^[^ ^234^, ^235^, ^236^, ^237^, ^238^ ^]^ More recently, stereoselective syntheses were developed to control conjugation and hence the corresponding properties^[^ ^213^, ^239^, ^240^, ^241^ ^]^ for applications in electroluminescent materials, stimuli‐responsive materials and dual‐state emitters.^[^ ^215^, ^216^ ^]^ The UV/vis absorption is typically red‐shifted with increasing chain lengths, in line with σ,π‐conjugation across the silicon centers, resulting in variable absorption bands ranging from *λ_abs_* = 240–500 nm and emission maxima between *λ_em_* = 350 and 620 nm. The absorption of thin films is generally even further red‐shifted due to π‐stacking.

These materials showed relatively high thermal stabilities (∼200–350 °C), according to thermogravimetric analyses (TGA). The incorporation of silyl or disilyl end groups in oligo(*p*‐phenylenevinylene)s into materials for laser applications was just recently shown to result in improved photostability and lower excitation energies for amplified spontaneous emission.^[^ ^242^ ^]^

**Scheme 8:**
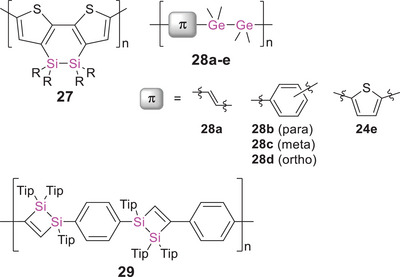
Representative examples of conjugated polymers with heavier tetrel–tetrel bridges **27**–**29** (R = *n*‐hexyl, Tip = 2,4,6‐triisopropylphenyl).

The Si─Si bonds can be cleaved under UV irradiation,^[^ ^222^, ^223^, ^234^, ^235^ ^]^ a feature that was used for the modification of TiO_2_ electrode surfaces employed in solar cells.^[^ ^179^, ^219^, ^233^ ^]^ For instance, poly(disilane) **27** (Scheme 8) and derivatives containing pyridine and pyrazine units as additional TiO_2_‐coordination sites were successfully attached to the electrode surfaces and proved applicable as dye‐sensitizing materials, albeit with low power conversion efficiencies of 0.16 to 0.89%.^[^ ^179^ ^]^ Thin films of poly(disilane)s become conductive upon preparation under an oxidizing atmosphere of SbF_5_ with conductivities up to 0.2 S cm^−1^, increasing to 1 S cm^−1^ when exposed to air.^[^ ^224^, ^225^, ^228^ ^]^

Germanium analogues **28a‐e** with vinylene, thiophene, and phenylene units (Scheme 8) showed hypsochromic shifts of the absorption maxima (*λ_abs_* = 240–260 nm) by Δ*λ* ∼ 10 nm, yet slightly higher conductivities (3–4 × 10^−4^ S cm^−1^) in comparison to the corresponding silicon congeners (1–2 × 10^−4^ S cm^−1^).^[^ ^243^, ^244^ ^]^ Preparing the films under iodine instead of SbF_5_ vapor results in slightly smaller values (∼1 × 10^−4^ S cm^−1^).

In 2014, Scheschkewitz et al. described the σ,π‐conjugated poly(1,2‐disilacyclobutene) **29** containing Si─Si units of which only one silicon is *directly* incorporated in the conjugation path while the other is in close proximity (Scheme 8).^[^ ^245^ ^]^ Effective conjugation of the π‐electrons across the low‐lying σ*‐orbitals at the bridging silicon centers was evident from a substantial bathochromic shift of the polymer absorption (*λ_abs_* = 310 nm) by Δ*λ* = 30 nm compared to the bis(1,2‐disilacyclobutene) monomer. DFT calculations additionally showed substantial contributions from the adjacent Tip_2_Si moiety to the LUMO. Poly(1,2‐disilacyclobutene) **25** is obtained by [2 + 2]‐cycloaddition of a *para*‐phenylene‐bridged tetrasilabutadiene^[^ ^246^ ^]^ and 1,4‐diethynylbenzene, one of the rare examples of a polymerization protocol employing heavier alkene homologues as polymerization precursors (further elaborated in Section 3).

### 2.3 Group 15: Recent Advances in Poly(pnictane)s

The incorporation of heavier Group 15 element motifs into π‐conjugated systems typically results in effective n,π‐ or σ,π‐conjugation, leading to a decrease of the LUMO energies and the HOMO‐LUMO gaps and hence in some cases to semiconducting properties. Additionally, the band gaps can be readily fine‐tuned by the coordination of electrophiles to the heteroelement and a concomitant change of the oxidation state. Accordingly, the corresponding polymers constitute promising materials for application in (opto‐)electronic devices, and indeed some poly(phosphole) and poly(arsole) derivatives have been employed in OLEDs, OSCs, and OFETs.^[^ ^42^, ^44^, ^45^, ^47^, ^97^, ^98^, ^99^, ^100^, ^101^ ^]^

**Figure 6:**
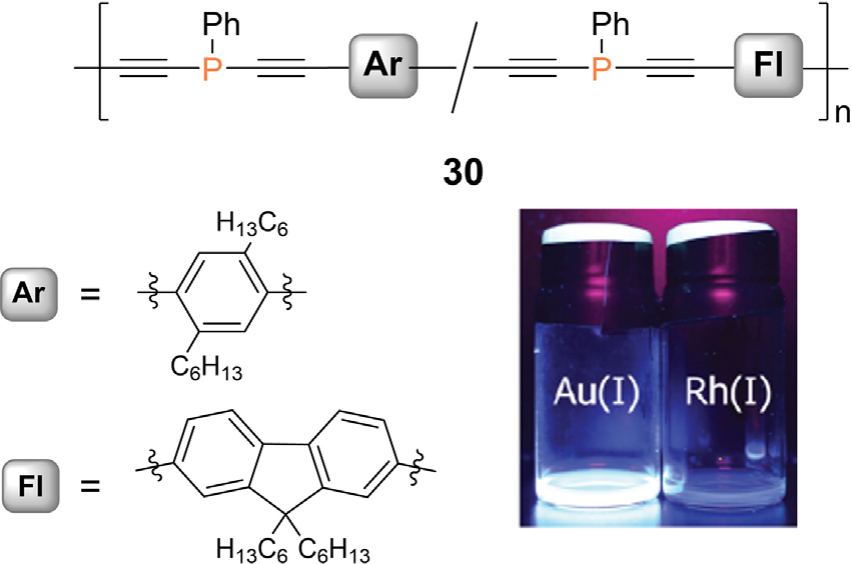
Fluorene‐ and phenylene‐bridged poly(diethynylenephosphane) statistical copolymer **30**. Solutions of a corresponding molecular model compound exhibiting blue fluorescence in the presence of Au(tht)Cl but not with [Rh(COD)Cl]_2_ (COD = cyclooctadiene). Adapted with permission from Ref. [249]. Copyright 2020 American Chemical Society.

In Lucht et al.’s first report on arylene‐bridged poly(phosphane)s, n,π‐conjugation across the phosphorus centers was indicated by a bathochromic shift (Δ*λ* = 15 nm) of the absorption (*λ_abs_* = 275–290 nm) in comparison to the monomer and by the appearance of additional weak maxima at higher wavelengths (*λ_abs_* = 415–435 nm).^[^ ^247^ ^]^ This was later corroborated by a systematic study of the electrochemical and optical properties of *p*‐phenylenephosphane aniline copolymers.^[^ ^248^ ^]^ Recently, Gates et al. reported an n,π‐conjugated statistical copolymer **30** (Figure 6), prepared by nickel‐catalyzed coupling of the two π‐bridged bis(alkynes) with phenyldichlorophosphane.^[^ ^249^ ^]^ The phosphorus atoms are incorporated into a π‐conjugated linear organic framework consisting of a random sequence of *para*‐phenylenediethynylene and fluorenyldiethynylene repeat units. In the presence of Au(I) and Au(III) (employed as Au(tht)Cl and HAuCl_4_·3H_2_O, respectively; tht = tetrahydrothiophene), highly blue‐fluorescent materials result: with Au(I), the corresponding polymeric complex **30**·(AuCl)_n_ with gold‐coordinated phosphorus centers was formed and in the case of Au(III), a presumably cross‐linked and thus insoluble polymer network was obtained.

**Figure 7:**
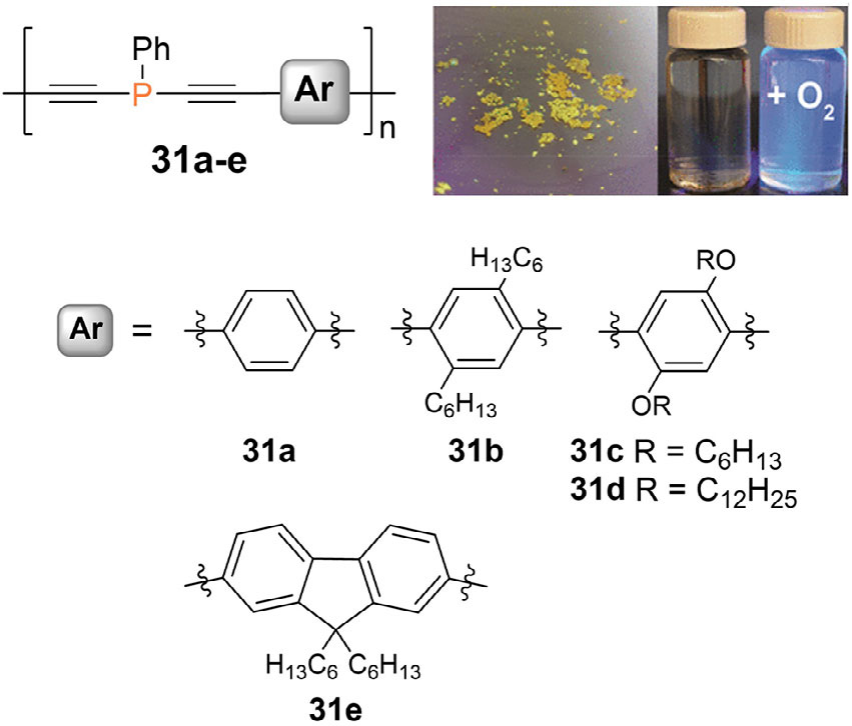
Poly(diethynylenephosphane) copolymers **31a–e** with different phenylene and fluorene spacers and the yellow solid‐state emission and blue solution emission after oxidation of a poly(phosphane). Adapted with permission from Ref. [251]. Copyright 2017 American Chemical Society.

The fluorescence was exclusively observed in the presence of gold ions but not with any other cation tested (alkali, earth alkali, and transition metals). Comparable sensing behavior had been reported earlier for different π‐conjugated phosphane‐diyne hybrid polymers **31a–e** (Figure 7), in which either a fluorene or a phenylene moiety is contained in the repeat unit.^[^ ^250^, ^251^ ^]^ The phosphorus atoms in **30** and **31a–e** are prone to oxidation in air or in the presence of H_2_O_2_, resulting in the formation of the corresponding phosphane oxide polymers, which show intense blue fluorescence in solution (*λ_em_* = 330–420 nm, *λ_abs_* = 290–410 nm) and weak yellow‐green fluorescence (*λ_em_* = 430–560 nm) in the solid state. The red‐shift of the thin film emission and the concomitant substantial broadening of the bands compared to the solution state are attributed to aggregate formation.

**Scheme 9:**
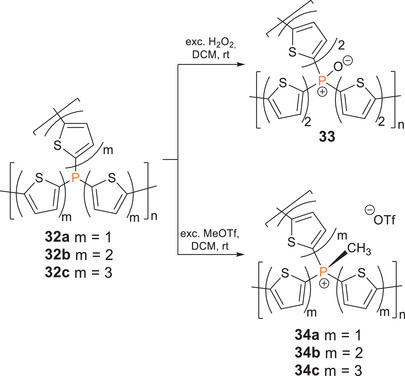
Post‐functionalization of phosphorus‐bridged thiophene polymer networks **32a–c** with H_2_O_2_ and MeOTf provides poly(phosphane oxide) **33** and methyl‐substituted cations **34a–c**.

Phosphorus‐bridged thiophene polymer networks **32a‐c** (Scheme 9) were obtained either by Stille P─C polycondensation of distannylthiophenes and PCl_3_ or by reaction of a dilithiated oligo(thiophene) with PCl_3_. Post‐functionalization of **32b** with H_2_O_2_ yielded phosphane oxide derivative **33** and reactions of **32a‐c** with MeOTf the methyl‐substituted cations **34a‐c** (*λ_abs_* = 500–800 nm). Methylated polymers **34b,c** were applied as photocatalysts for the hydrogen evolution reaction. Increasing conversion rates from the phosphane catalyst (155 µmol h^−1^ g^−1^) and the phosphane oxide (900 µmol h^−1^ g^−1^) to the cationic methyl‐substituted phosphane (2050 µmol h^−1^ g^−1^) confirm a strong dependency of the catalytic activity on the nature of the phosphorus centers and hence showcase the possibilities for tailoring polymer properties according to specific requirements through heteroatom incorporation and post‐functionalization.^[^ ^252^ ^]^

As demonstrated by the “turn‐on” fluorescence of the phosphane oxide polymers by Gates et al. (vide supra),^[^ ^250^, ^251^ ^]^ compounds with oxidized P(V) centers are particularly interesting, due to even lower LUMO levels than the corresponding phosphanes.

**Figure 8:**
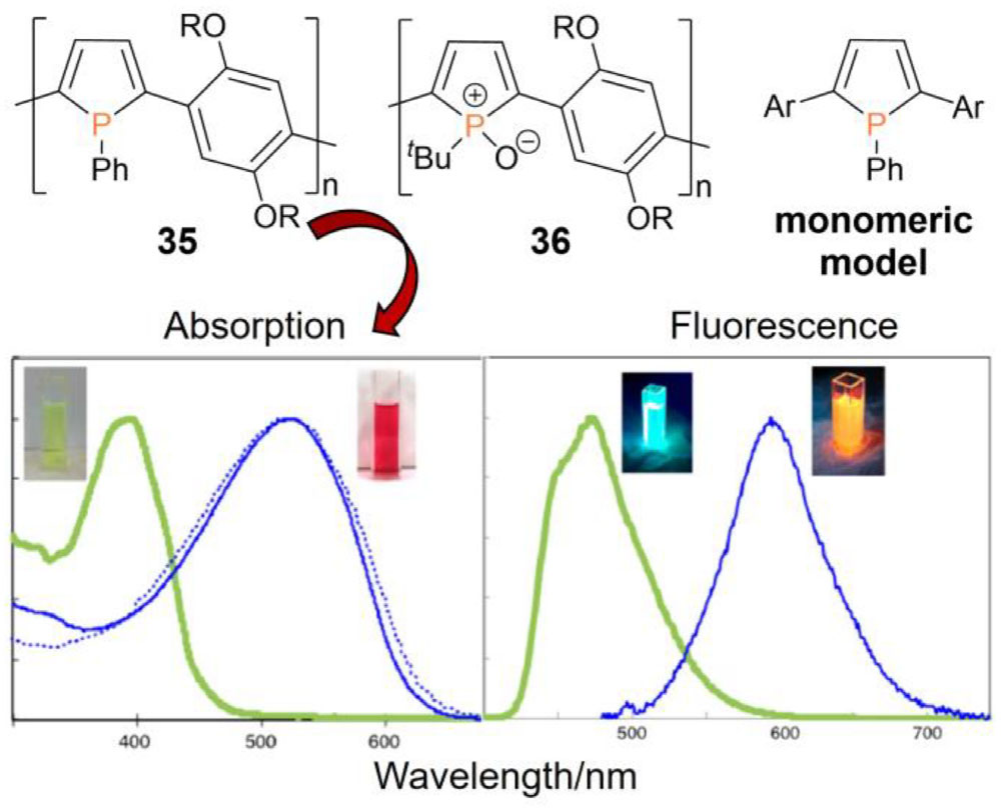
Top: Structure of phenylene‐bridged poly(phosphole) **35**, *tert*‐butyl‐substituted poly(phosphole oxide) **36** and a monomeric model compound (R = 2‐ethylhexyl, Ar = 2‐methoxyphenyl). Bottom: Absorption and emission spectra of polymer **35** (blue lines) show huge bathochromic shifts compared to the monomeric model compound (green lines). Adapted with permission from Ref. [253]. Copyright 2015 American Chemical Society.

Tomita et al. prepared the phosphole‐phenylene copolymer **35** and the oxidized analogue **36** (Figure 8) by reactions of the corresponding titanacyclopentadiene polymer with PhPCl_2_ and *^t^* BuPCl_2_, respectively.^[^ ^253^ ^]^ While the phenyl‐substituted derivative **35** proved stable in air, oxidation to *tert*‐butyl‐substituted **36** occurred during workup. Both polymers exhibit orange to yellow fluorescence. Comparison of the photophysical properties of the phenyl‐substituted poly(phosphole) **35** with a monomeric model compound (Figure 8) revealed huge bathochromic shifts of the absorption (*λ_abs_* = 522 nm) and emission maxima (*λ_em_* = 594 nm) by Δ*λ* = 120–130 nm due to effective π‐conjugation along the chain. Notably, *tert*‐butyl derivative **36** shows a substantial hypsochromic shift in comparison (*λ_abs_* = 445 nm, *λ_em_* = 547 nm), which was not discussed in detail by the authors, but most probably arises from a more effective lowering of the LUMO by the phosphorus in **35** with the electron‐withdrawing phenyl substituent.

**Figure 9:**
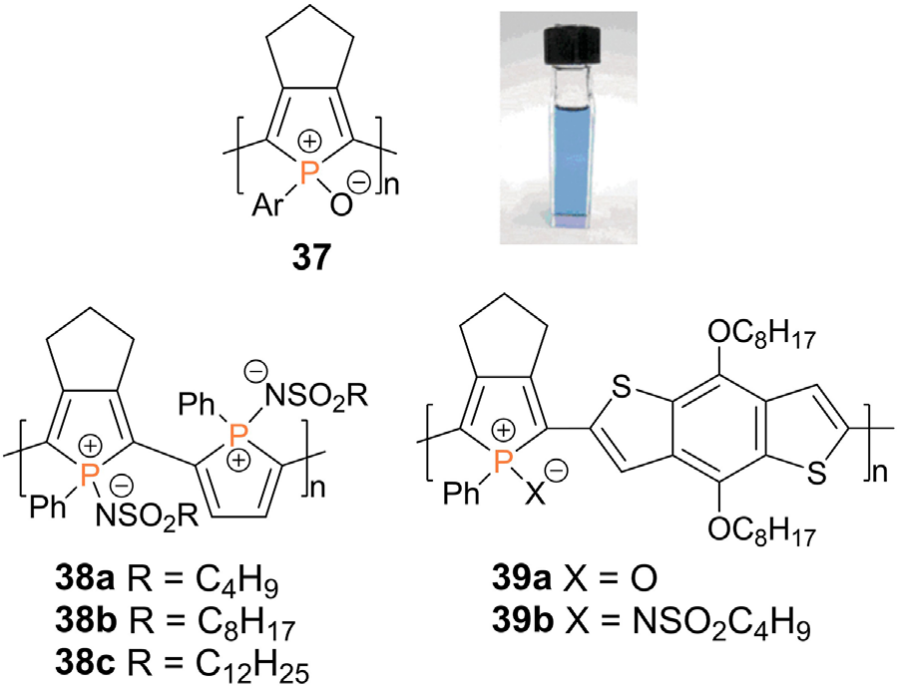
Structurally related poly(phosphole oxide)s and poly(phosphole imide)s **37**–**39** (Ar = *p*‐dodecyloxyphenyl). Solution of poly(phosphole) **37**. Adapted with permission from Ref. [254]. Copyright 2010 American Chemical Society.

In contrast to **36**, poly(phosphole oxide) **37** (Figure 9) was obtained in a Stille coupling reaction of the distannyl and the diiodo phosphole oxides in a 1:1 ratio.^[^ ^254^ ^]^ The UV/vis absorption (*λ_abs_* = 655 nm) shows an even larger red‐shift by Δ*λ* = 270 nm in comparison to the corresponding monomer *(λ_abs_* = 386 nm). The weak fluorescence, observed in the monomer, dimer, and trimer, is shifted from blue‐green to orange with increasing chain length and even into the NIR region in the case of the polymer **37**. The absorption maximum of the aryl‐substituted oxidized poly(phosphole) resides in the far red of the visible spectrum—in line with a blue appearance (Figure 9)—and is hence substantially bathochromically shifted (Δ*λ* = 130 nm) compared to that of phenyl‐substituted poly(phosphole) **35** (*λ_abs_* = 522 nm).

Poly(phosphole imide)s **38a‐c** with alkylsulfonylimino‐substituted phosphorus atoms (Figure 9) provide an even more distinct decrease of the LUMO levels and smaller HOMO‐LUMO gaps with absorption bands reaching into the NIR region.^[^ ^255^ ^]^ Considerable red shifts of the absorption maxima (*λ_abs_* = 670–680 nm) in comparison to a corresponding phosphole imide monomer (*λ_abs_* = 400 nm) demonstrate efficient π‐conjugation along the polymer chains. The resulting charge carrier mobilities (up to 6·10^−3^ cm^2^ V^−1^ s^−1^) are suitable for use as semiconducting materials; longer alkyl substituents at the sulfonyl group showed a slightly enhancing effect. Donor–acceptor copolymers **39a,b**, with electron‐rich benzodithiophene units in combination with the phosphole oxide moieties, exhibit a red‐shifted absorption (*λ_abs_* = 580–600 nm) in solutions and in thin films and have been employed in OSCs.^[^ ^256^ ^]^ A slightly improved performance is achieved with the oxide (**39a**) in comparison to the imide (**39b**), presumably due to a less bulky environment and hence stronger intermolecular π‐π interactions.

**Figure 10:**
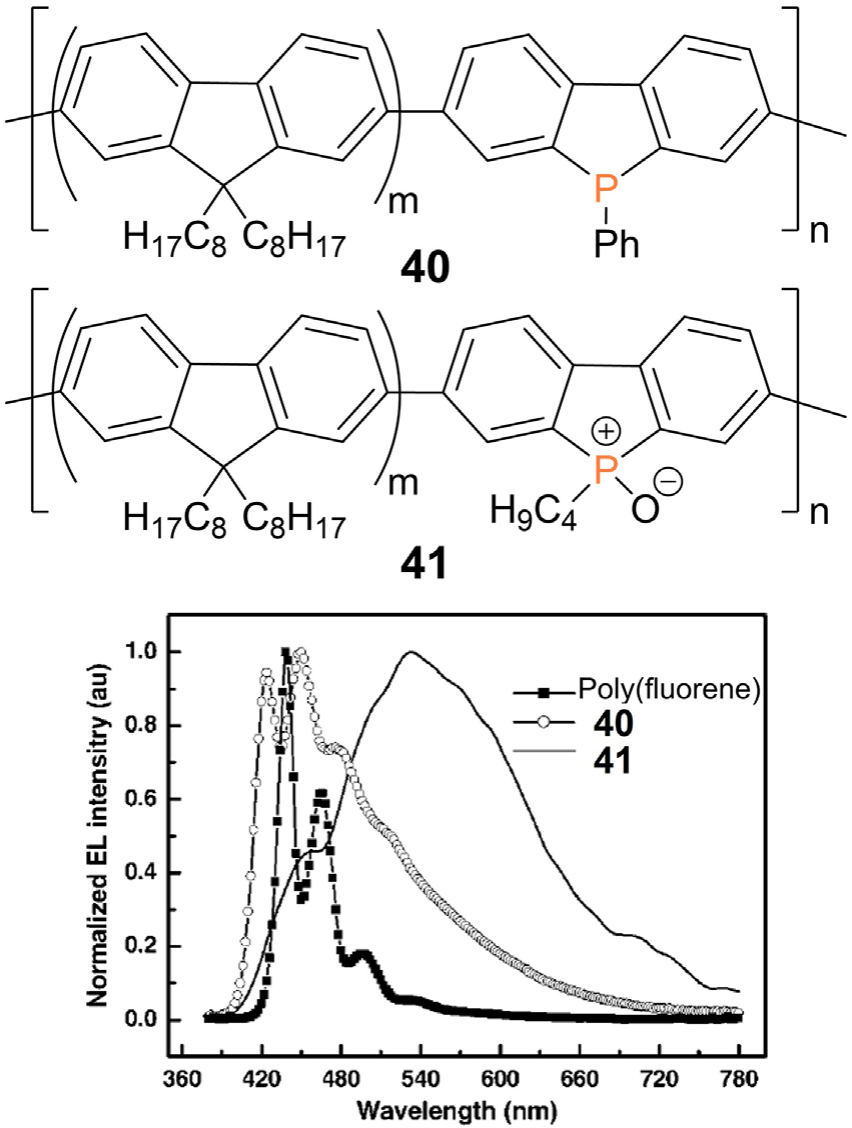
Poly(phosphafluorene) **40** gives rise to blue light emission in an OLED device, while the oxidized poly(phosphafluorene oxide) **41** exhibits white light emission under the same conditions. Fluorescence spectra adapted with permission from Ref. [257]. Copyright 2008 American Chemical Society.

Considerable differences in the emission behavior due to different oxidation states of the phosphorus atoms in the polymer chain have also been observed in a comparative study of the electroluminescence in OLEDs.^[^ ^257^ ^]^ The emissive layers consisted either of poly(phosphafluorene) **40** or its oxidized form **41**, both obtained by Suzuki coupling of the corresponding fluorene and heterofluorene comonomers. P(V) containing polymer **41** with a smaller optical band gap (2.74 eV) compared to **40** (2.81 eV) gives rise to white‐light emission, while **40** with P(III) moieties exhibits intense blue emission with maxima at *λ_em_* = 424, 450, and 478 nm (Figure 10).

In thin films of poly(oxophosphafluorene)s with phenylene spacers, intense green‐blue fluorescence (*λ_em_* = 460–470 nm) was also observed.^[^ ^258^ ^]^ The longest wavelength absorptions of **40** and **41** differ only slightly (**40**: *λ_abs_* = 384 nm, **41**: *λ_abs_* = 402 nm) and exhibit additional shoulders at 429 and 430 nm, respectively, attributed by the authors to a β‐phase with increased co‐planarity.^[^ ^257^ ^]^

The incorporation of benzodithiophene units in poly(phosphafluorene)s with either oxo or sulfur functionalities at the phosphorus centers, on the other hand, results in a bathochromic shift of the absorption maxima (*λ_abs_* = 465, 466 nm), which is even more pronounced (*λ_abs_* = 530 nm) in a gold(I)‐coordinated poly(phosphafluorene).^[^ ^259^ ^]^ Employed in OSC devices, these post‐functionalized benzodithiophene phosphafluorene copolymers resulted in low, slightly variable power conversion efficiencies (O: 0.13%, S: 0.60%, Au: 0.26%).

**Scheme 10:**
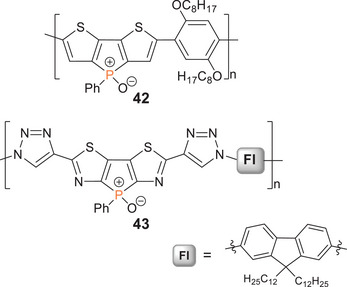
Representative examples of poly(phosphole oxide)s **42** and **43** with fused heterole substituents.

Poly(dithienophosphole oxide) **42** with phenylene bridging units (Scheme 10) was obtained by Stille coupling of the distannylphosphole oxide monomer and *p*‐diiodo‐2,5‐dioctyloxybenzene and showed yellow‐green luminescence (*λ_em_* = 555 nm), considerably red‐shifted compared to the dithienophosphole oxide monomer (*λ_em_* = 463 nm).^[^ ^260^ ^]^ Poly(dithiazolophosphole oxide) **43** with triazole‐bridged fluorene units (Scheme 10) was obtained by Huisgen cycloaddition of the corresponding fluorenyl‐diazide and the phosphole with two ethynylene substituents.^[^ ^261^ ^]^ The chain growth is presumably limited by poor solubility and thus only short oligomers of **43** were obtained. Benzodithiophene copolymers of dithienophosphole oxides were employed in OSCs providing relatively high‐power conversion efficiencies of 6%–7%.^[^ ^262^ ^]^

**Scheme 11:**
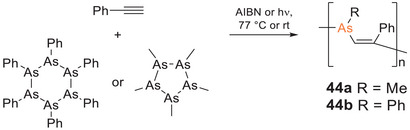
Free‐radical copolymerization of cycloarsanes and phenylacetylene yielded the first arsenic‐embedded conjugated polymers **44a,b** (AIBN = Azobisisobutyronitrile).

Conjugated polymers with As(III) centers in the main chain should show a lower tendency toward oxidation compared to their phosphorus analogues. Poly(arsane)s have only come to the fore during the last decade,^[^ ^102^ ^]^ although the first examples had already been reported by Chujo and Naka et al. as early as 2002: the alternating poly(vinylenearsane)s **44a,b** were obtained selectively by free‐radical copolymerization of pentamethylcyclopentaarsane or hexaphenylcyclohexaarsane with phenylacetylene (Scheme 11).^[^ ^263^ ^]^ Phenyl‐substituted **44b** is a colorless solid, whereas the methyl‐substituted polymer **44a** appears bright yellow. Although the differences between the optical properties of **44a** and **44b** were not discussed explicitly by the authors, the yellow color of **44a** – in line with the absorption tailing into the visible range up to *λ_abs,onset_* = 550 nm—was attributed to n → π* transitions along the polymer chain. Furthermore, **44a** exhibits green‐blue luminescence (*λ_em_* = 485 nm) with maximum intensity at an excitation wavelength of *λ_exc_* = 396 nm. In stark contrast to related phosphorus compounds (vide supra), ^1^H NMR spectroscopy suggests poly(vinylenearsane) **44a** to be stable toward oxidation, even in the presence of H_2_O_2_.

**Scheme 12:**
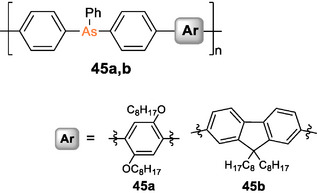
Poly(triphenylarsane)s **45a,b**.

Most recently, trivalent arsane motifs have been embedded into the π‐conjugation path of phenylene and fluorene groups to result in **45a,b** (Scheme 12).^[^ ^264^ ^]^ The n,π‐conjugated polymers were obtained by Suzuki–Miyaura polycondensation of *para*‐dibrominated triphenylarsane with the corresponding bis(boronic acid) comonomer. DFT calculations confirmed that the arsane moiety contributes considerably to a decrease of the LUMO energy. The observed bathochromic shift of the absorption (*λ_abs_* = 335, 340 nm) by Δ*λ* ∼ 50 nm compared to a model compound of the repeat units was, however, shown to be mainly caused by an extension of the π‐system across the phenyl rings rather than across the arsenic atoms. Blue emission (*λ_em_* = 405, 412 nm) with considerable quantum yields (17%–65%) was observed for both **45a,b**, as well considerably red‐shifted by Δ*λ* ∼ 60 nm compared to the monomeric model system.

**Figure 11:**
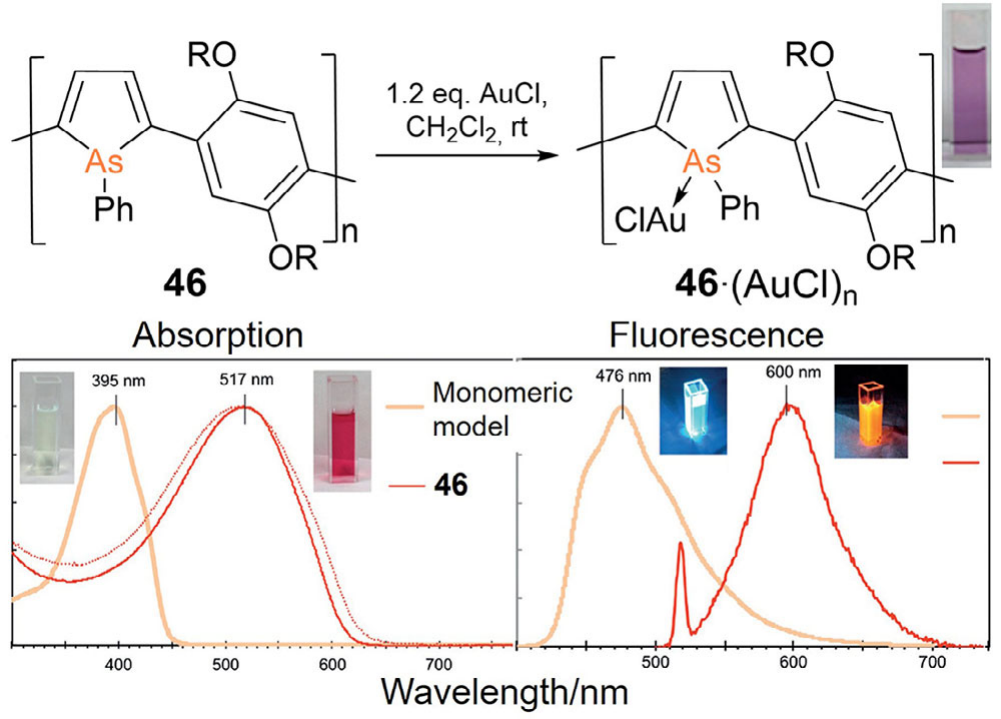
Top: Reaction of poly(arsole) **46** with AuCl (R = 2‐ethylhexyl). Bottom: Solutions of **46** and **46**·(AuCl)_n_ under daylight and upon UV irradiation as well as the absorption and fluorescence spectra. Adapted with permission from Ref.[266]. Copyright 2016 Wiley VCH.

The first examples of arsole‐based polymers were reported in 2016 independently by the groups of Heeney, Naka and Tomita.^[^ ^265^, ^266^, ^267^ ^]^ Tomita et al. synthesized arsole‐phenylene copolymer **46** (Figure 11) by transmetalative post‐functionalization of the corresponding titanacyclopentadiene polymer,^[^ ^266^ ^]^ a strategy they had already implemented in the syntheses of Group 14 cyclopentadiene polymers and poly(phosphole) derivatives (vide supra). The employed diiodoarsane reagent was generated from hexaphenylcyclohexaarsane in a reaction with iodine.^[^ ^268^ ^]^ Poly(arsole) **46** precipitates from methanol as a dark red solid with similar absorption and fluorescence properties (*λ_abs_* = 517 nm, *λ_em_* = 600 nm) as its phosphorus analogue **35** (*λ_abs_* = 522 nm, *λ_em_* = 594 nm).^[^ ^253^ ^]^

Polyarsole **46** exhibits quasi‐reversible oxidation peaks in cyclic voltammetry, whereas the corresponding poly(phosphole) **35** is irreversibly oxidized under the same conditions. In analogy to Gates’ poly(phosphane) **30** (vide supra),^[^ ^249^ ^]^ poly(arsole) **46** forms the corresponding **46**·(AuCl)_n_ polymer with Au(I)‐coordinated arsenic centers. The LUMO level and thus the HOMO‐LUMO gap are lowered, as experimentally confirmed by bathochromic shifts of Δ*λ* = 30 nm for both, absorption and emission.

**Figure 12:**
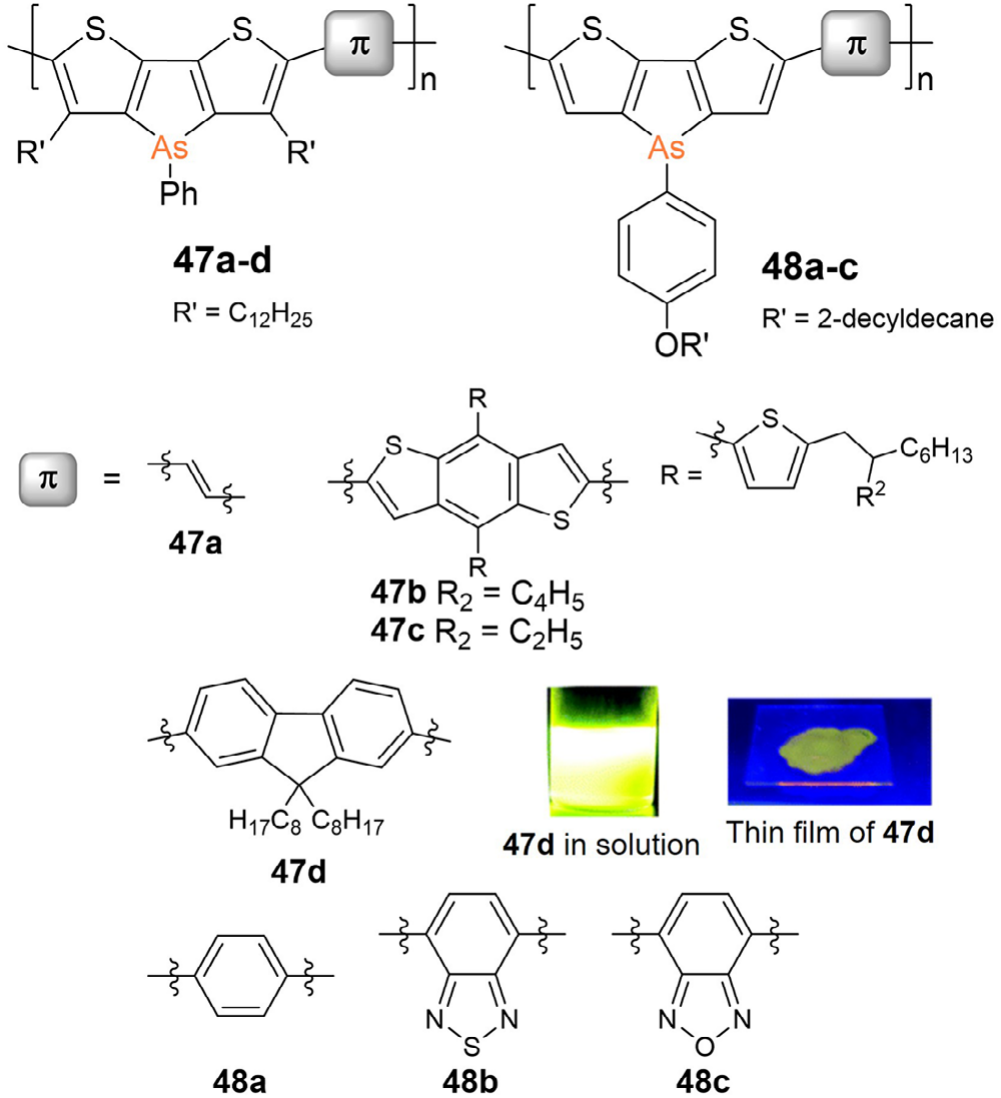
Poly(dithienoarsole)s **47a–d** with solubility‐enhancing alkyl groups in the 3‐position of the thiophene rings and **48a–c** with *para*‐alkoxyphenyl substituents at the arsenic centers. Images of the yellow fluorescence of **47d** in solution and in the solid state. Adapted with permission from Ref. [267]. Copyright 2016 The Royal Society of Chemistry.

Heeney et al. reported the synthesis of the first poly(dithienoarsole) **47a** (Figure 12).^[^ ^265^ ^]^ The dark blue vinylene‐containing copolymer with a remarkably high absorption at longest wavelength of *λ_abs_* = 616 nm was employed in an OFET and exhibited promising charge carrier mobilities. Subsequently, the corresponding poly(dithienoarsole)s **47b,c** with benzodithiophene units were synthesized and applied to OSC devices with reasonable performance.^[^ ^269^ ^]^ Poly(dithienoarsole) **47d** with bridging fluorene units was obtained by Suzuki–Miyaura polycondensation,^[^ ^267^ ^]^ whereas **47a‐c** were synthesized by Stille cross‐coupling. In the case of **47d**, a broad absorption band with a maximum at *λ_abs_* ∼ 450 nm and a shoulder of almost equal intensity at ∼520 nm was observed. The intense yellow fluorescence of a solution of **47d** (*λ_em_* ∼ 550 nm, Figure 12) is slightly red‐shifted but weakened in thin films (*λ_em_* ∼ 600 nm).

Similar red‐shifts were observed for the thin film absorption of poly(arsole)s **47a–c**, presumably due to aggregation of the π‐systems. This interpretation is supported by the absence of such a red‐shift upon deposition in a poly(methylmethacrylate) (PMMA) matrix. In comparison to molecular model compounds, the fluorescence of **47d** showed a large bathochromic shift by Δ*λ* ∼ 150 nm (Figure 12) as a result of effective extension of the conjugated system along the polymer chain. Due to the remarkably high fluorescence quantum yield of **47d** (44%), and its low tendency toward oxidation, it was applied as a photooxidation catalyst and as highly efficient photosensitizer for singlet oxygen generation.^[^ ^270^ ^]^ Recent progress was achieved through solubility‐enhancing formal *para*‐alkoxylation of the phenyl substituent at the arsenic centers of poly(dithienoarsole)s **48a–c**, rendering the incorporation of sterically demanding long alkyl chains in 3‐position of the thiophene rings (R′ in **47a–d**) redundant.^[^ ^271^ ^]^ This allowed for copolymerization with benzothiadiazole and benzoxadiazole as electron acceptor units in highly co‐planar donor‐acceptor copolymers **48b,c**. The resulting decrease of the LUMO levels caused red‐shifted absorptions (**48b**: *λ_abs_* = 682 nm, **48c**: *λ_abs_* = 664 nm) and huge Stokes shifts (Δ*λ* = 160–230 nm) to NIR emission with maxima at *λ_em_* = 840 (**48b**) and 886 nm (**48c**).

**Scheme 13:**
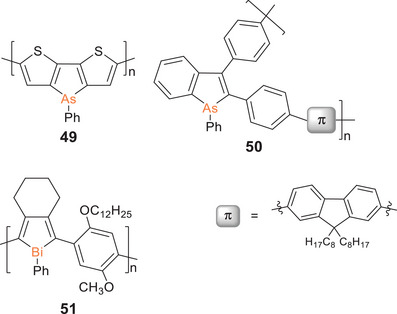
Poly(dithienoarsole) **49**, 2,3‐substituted poly(arsole) **50** (substitution along the polymer chain is regiorandom; for clarity, only one representative constitution of the repeat unit is depicted) and the only reported poly(bismole) **51**.

Dithienoarsole homopolymer **49** without additional π‐linking units (Scheme 13) was obtained as an insoluble red‐colored thin film on an indium tin oxide electrode by electropolymerization of the corresponding dithienoarsole monomer.^[^ ^272^ ^]^ Intense absorption maxima were observed in the UV/vis spectrum of the polymer film at *λ_abs_* = 387 and 462 nm as well as a weaker red‐shifted band (*λ_abs_* = 630 nm) comparable to the absorption of vinylene copolymer **47a** (*λ_abs_* = 616 nm) but at considerably higher wavelengths than the corresponding benzodithiophene and fluorene copolymers **47b–d** and arylene‐bridged poly(arsole) **46** (*λ_abs_* = 450–520 nm).

An alternating arsafluorene‐fluorene copolymer, obtained in an analogous manner to the poly(dithienoarsole)s by Suzuki–Miyaura polycondensation, exhibits an absorption maximum further in the blue (*λ_abs_* = 387 nm) and shows amplified spontaneous emission (*λ_em_* = 458 nm), crucial for the use in photonics, such as lasers.^[^ ^273^, ^274^ ^]^ Recently, poly(arsole) **50** with 2,3‐connectivity of the arsole rings (Scheme 13) was reported to exhibit intense green luminescence (*λ_em_* = 500 nm).^[^ ^275^ ^]^ This observation and the red shift in comparison to monomeric model compounds (*λ_em_* = 460–490 nm) suggest considerable influence of the arsane unit on the frontier orbitals of the conjugated system in this bent regioisomer as well.

While there are no antimony‐based hybrid polymers at present, the first example of a conjugated polymer with bismuth directly adjacent to the conjugation path by Chujo et al.^[^ ^276^ ^]^ is in line with the general trend toward the heavier elements as seen in Group 13 and 14. Poly(bismole) **51** (Scheme 13) exhibits green‐blue emission (*λ_em_* ∼ 440 nm, *λ_abs_* = 311 nm) and was synthesized from a poly(zirconacyclopentadiene) by post‐functionalization via lithiation and subsequent reaction with BiPhBr_2_.

## 3 Diheteroatomic Multiple Bonds Embedded in the Main Chain

### 3.1 Group 13: B═N Bridges in Conjugated Hybrid Polymers & a Poly(phosphaborene)

The zwitterionic B═N moiety is associated with a much larger HOMO–LUMO gap than corresponding C═C bonds, rendering poly(iminoborane)s air‐stable materials with a priori dielectric rather than (semi‐)conducting properties. The increased band gap creates new possibilities regarding the fine‐tuning of the electronic properties of resulting materials for the use in optoelectronic devices, for instance, through combination with organic polymers.^[^ ^277^, ^278^ ^]^ Numerous examples of poly(iminoborane)s with B═N units in the main chain have been reported in recent years.^[^ ^278^ ^]^

**Scheme 14:**
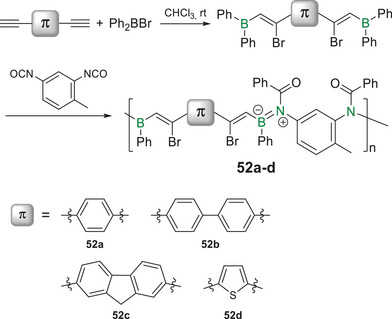
Representative examples of poly(iminoborane)s **52a–d** with variable bridging units.

Chujo et al. reported on the syntheses of the first poly(iminoborane)s, applying diisocyanate monomers, readily available building blocks of ubiquitous poly(urethane)s.^[^ ^279^, ^280^ ^]^ Phenylene‐bridged derivatives with acyl groups at the nitrogen atoms are either obtained by alkoxyboration of a diisocyanate with mesityl dimethyl boronate at 150 °C^[^ ^281^ ^]^ or as copolymers **52a–d** in a two‐step reaction (Scheme 14)^[^ ^282^, ^283^ ^]^: In the first step, 1,4‐diethynylbenzene undergoes haloboration with Ph_2_BBr at room temperature, followed by phenylboration of the subsequently added diisocyanate in the second step. Notably, **52d** with thiophene bridges exhibits green fluorescence in solution. The anticipated extension of the conjugation path length along the polymer chain was, however, not confirmed experimentally, which is not entirely surprising, regarding the highly polar character of the B═N bond. To illustrate this point: in borazine, the so‐called “inorganic benzene”, the electrons are as well mainly localized at nitrogen, resulting in only marginal ring currents, in sharp contrast to the well‐known aromatic benzene.^[^ ^284^, ^285^, ^286^ ^]^ It should, however, be mentioned that a recent computational analysis found that the CH bridges between the BN units in carborazine reduce the effective electronegativity difference and hence account for aromaticity comparable to benzene.^[^ ^287^ ^]^ Nonetheless, the electronic structure of the *syn*‐regioregular azaborine polymer reported by Jäkle and Liu et al. resembles that of the cyclohexadiene rather than the phenylene analogue.^[^ ^288^ ^]^ It exhibits considerably red‐shifted absorption (*λ_abs_* = 457 nm) compared to the monomer (*λ_abs_* = 277 nm) and, in contrast to the latter, orange fluorescence (*λ_em_* = 600 nm). Thiophene‐bridged derivatives were employed in an OFET with hole mobilities of up to 0.38 cm^2^ V^−1^ s^−1^.^[^ ^289^ ^]^

**Scheme 15:**
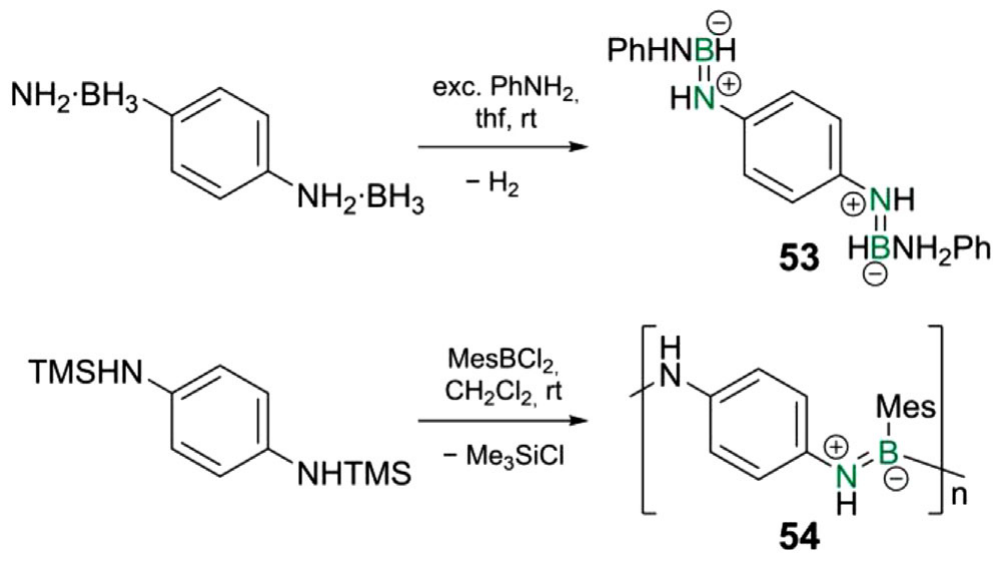
Spontaneous dehydrocoupling of the bis(borane) adduct of *para*‐phenylenediamine to bis(iminoborane) **53** and Si/B exchange polycondensation of disilyl‐substituted *para*‐phenylenediamine with dichloromesitylborane to poly(iminoborane) **54** (TMS = trimethylsilyl, Mes = 2,4,6‐trimethylphenyl).

In recent years, Helten et al. investigated potentially π‐conjugated linear polymers containing B═N units in the main chain. The bis(borane) adduct of *para*‐phenylenediamine was found to undergo spontaneous dehydrocoupling in the presence of aniline to provide the dimer **53** with NBN units bridged by *para*‐phenylene groups (Scheme 15).^[^ ^290^ ^]^ Dissolution of the mono(borane) adduct of *para*‐phenylenediamine in thf results in gas evolution as well, indicating the formation of dihydrogen and presumably the corresponding polymer, which, however, turned out to be insoluble and eluded further characterization. Solubility enhancing mesityl groups at the boron centers were incorporated by using dichloromesitylborane in a Si/B metathesis with *N*,*N*′‐bis(silyl)‐substituted *para*‐phenylenediamine. Subsequent end‐capping with 4‐*tert*‐butyl‐*N*‐trimethylsilylaniline provided poly(iminoborane) **54** with a degree of polymerization of *X_n_* = 33 (Scheme 15).

The sterically demanding mesityl group induces a distinct preference for the *E*,*Z*‐configuration according to DFT calculations and in line with the crystal structure of the corresponding monomeric diaminoborane. Along the polymer chain of **54**, however, different configurations are encountered according to the observed splitting of characteristic ^1^H NMR signals upon lowering the temperature and in agreement with DFT calculations on the dimer. The longest wavelength absorptions of dimer **53** and polymer **54** are both observed in the UV at *λ_abs_* = 290 and 295 nm, respectively, and only slightly bathochromically shifted by Δ*λ* = 20–25 nm compared to those of the monomers. This was taken as an indication of extended π‐conjugation, although TD‐DFT calculations on molecular model compounds additionally confirmed some extent of charge transfer to the boron. The delocalization across the phenylene linker, the B═N moieties and the terminal phenyl groups was supported by the topologies of HOMO and LUMO according to DFT calculations. Considerable deviations from coplanarity, however, imply a significantly diminished π‐conjugation along the polymer chain. The monomeric, oligomeric and polymeric compounds exhibit blue fluorescence (*λ_em_* = 420–470 nm) with large Stokes shifts (Δ*λ* = 160 nm in the case of polymer **54**), suggesting the relaxation of twisted intramolecular charge transfer excited states and thus emphasizing the participation of the boron centers along the chain rather than explicit π‐conjugation.

**Figure 13:**
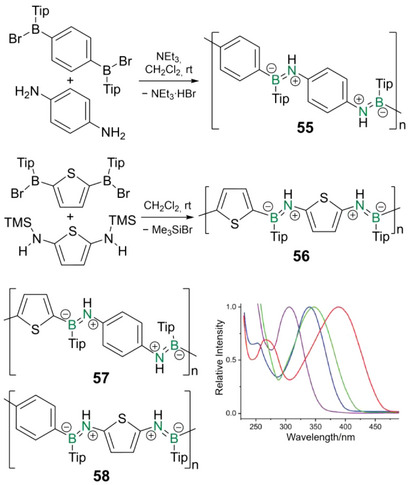
Top: HBr and Me_3_SiBr elimination polycondensation methods for the synthesis of iminoborane analogues of poly(*p*‐phenylenevinylene) **55** and poly(thiophenevinylene) **56** (Tip = 2,4,6‐triisopropylphenyl, TMS = trimethylsilyl). Bottom: Poly(iminoborane)s **57** and **58** with alternating thiophene and phenylene units and absorption spectra of the all‐thiophene derivative **56** (red line), the corresponding monomeric system (purple line) and regioisomers of the dimeric models (blue and green lines). Adapted with permission from Ref. [293]. Copyright 2023 Wiley VCH.

Iminoborane analogues of poly(*p*‐phenylenevinylene) (PPV) and poly(thiophenevinylene) **55–58**, were obtained by two conceptually related strategies (Figure 13). Homopolymer **55** and copolymer **57** can be prepared by both methods, which involve either HBr or TMSBr (TMS = trimethylsilyl) elimination from *para*‐phenylenediamine or the corresponding *N*‐silylated derivative on the one hand side and the appropriate bis(bromoboryl) species on the other.^[^ ^291^, ^292^ ^]^

In contrast, the thiophene homopolymer **56** and copolymer **58** are only accessible via the Si/B metathesis route due to the instability of the silyl‐free diaminothiophene precursor.^[^ ^292^, ^293^ ^]^ The bromide end groups in the resulting polymers were subsequently exchanged with dimethylamino groups through addition of TMSNMe_2_. Degrees of polymerization between *X_n_* = 19 and 70 were obtained, with a slight trend toward higher values in the case of the HBr elimination pathway. Poly(iminoborane)s **55**–**58** give rise to remarkable bathochromic shifts of Δ*λ* = 60 to 80 nm of the absorption (**55**: *λ_abs_* = 340 nm, **56**: *λ_abs_* = 390 nm, **57**: *λ_abs_* = 360, **58**: *λ_abs_* = 370 nm) in comparison to the respective monomers (Figure 13). This finding was interpreted as evidence for extended π‐conjugation across the B═N double bonds along the polymer chain. The shifts are considerably larger than in the case of poly(diaminoborane) **54** (vide supra), possibly due to the incipient donor‐acceptor character in copolymers **55–58**, promoting charge‐transfer along the chain. This was particularly emphasized in the case of the aminothiophene‐borylbenzene copolymer **58**: corresponding molecular model compounds with a similar substitution pattern showed a slight tendency toward higher wavelength absorptions in comparison to the corresponding aminophenylene‐borylthiophene derivatives.

In line with favorable orbital overlap between the organic framework and the B═N units and hence substantial electron transfer, the crystal structures of the monomeric model systems exhibit coplanarity of the π‐substituents and the B═N units, which is, however, slightly less pronounced for the dimers. The frontier orbitals of dimers of **55**–**58** clearly indicate charge transfer from the π‐spacer, where the HOMO is located, to the terminal π‐ligands, showing major contributions to the LUMO. Systematic red‐shifts of the absorption and the fluorescence (in PMMA films) are observed with increasing thiophene content: the all‐thiophene polymer **56** exhibits yellowish‐green luminescence (*λ_em_* = 521 nm), whereas the all‐phenylene derivative **55** shows deep‐blue fluorescence (*λ_em_* = 450 nm). The alternating copolymers **57** (*λ_em_* = 460 nm) and **58** (*λ_em_* = 502 nm) are characterized by emissions in between those of **55** and **56**, with a tendency toward higher wavelengths for **58** with favorable donor‐acceptor matching: the donor strength of the nitrogen center is enhanced by the adjacent thiophene and the electron‐deficiency of boron by the moderately electron‐accepting phenylene linker. Striking hypsochromic shifts of the emission and a substantial intensity increase were observed for solutions of the corresponding dimers with increased water content, strongly suggesting aggregation‐induced emission. Indeed, dynamic light scattering (DLS) measurements of the dimers confirmed the presence of particles with hydrodynamic radii between 130 and 160 nm. A positive solvatochromic effect due to intramolecular charge transfer upon excitation was presumed to counteract the hypsochromic shift, almost canceling it out in the case of the aminothiophene‐containing dimers of **56** and **58**. In order to shed more light on the impact of the charge transfer due to the B═N moieties, the implementation of more electrophilic acceptor units, for instance, the well‐established benzothiadiazole, might be a promising next step. As a consequence, the multiple bond character of the B═N bonds should be increased and charge‐transfer along the polymer chain promoted, with implications for the absorption and emission.

**Figure 14:**
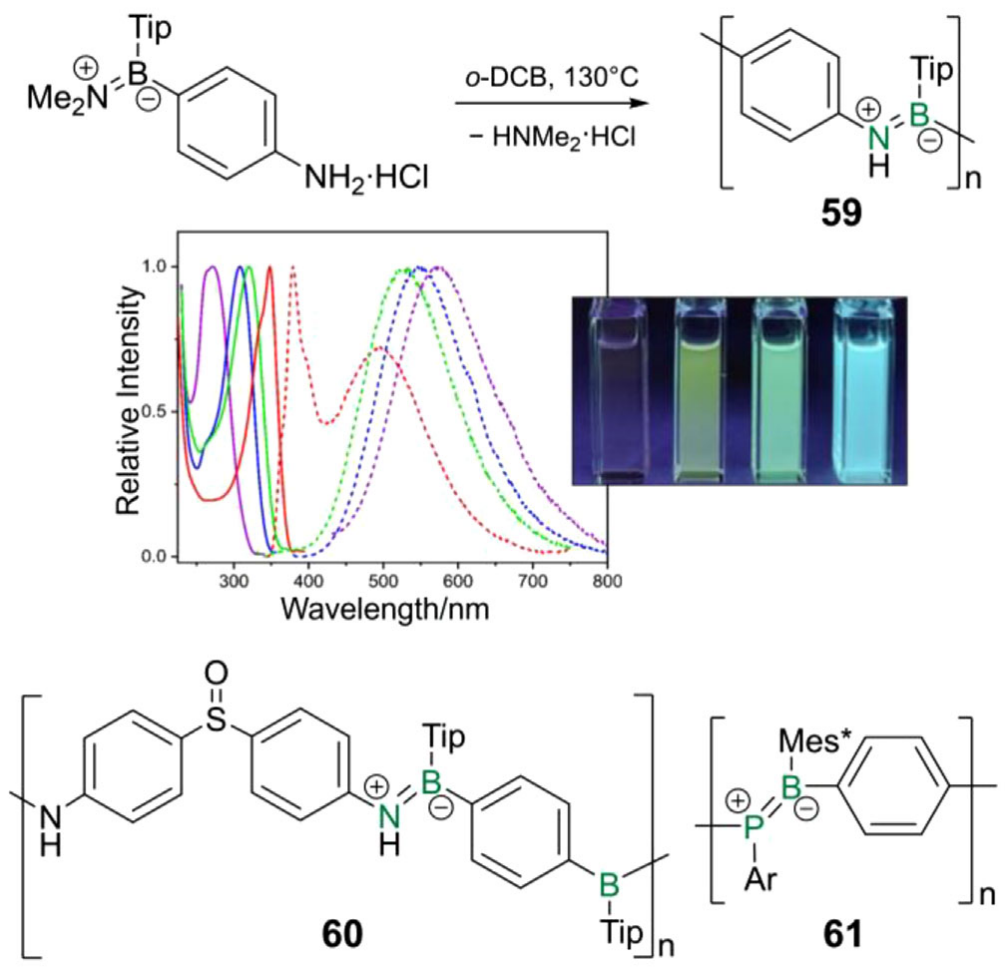
Top: Polymerization of a *para*‐aminophenylene iminoborane at elevated temperature providing poly(iminoborane) **59**. Absorption (solid lines) and fluorescence spectra (dotted lines) of **59** (red) and of the corresponding monomer (purple), dimer (blue) and trimer (green) and their solutions under UV light irradiation. Adapted with permission from Ref. [294]. Copyright 2025 Wiley VCH. Bottom: Poly(iminoborane) **60** with sulfoxide bridging units and poly(phosphaborene) **61** (oDCB = *ortho*‐dichlorobenzene, Tip = 2,4,6‐triisopropylphenyl, Mes* = 2,4,6‐tri‐*tert*‐butylphenyl, Ar = 2,6‐dimethyl‐4‐octylphenyl).

Very recently, the same group developed a poly(iminoborane) **59** with a strictly alternating BN sequence (Figure 14), as opposed to the previous examples with BN/NB alternation.^[^ ^294^ ^]^ Polymer **59** was prepared by heating a solution of a *para*‐aminophenylenedimethyliminoborane for several days under the release of dimethylamine. The UV/vis absorption is bathochromically shifted with increasing chain length (monomer: *λ_abs_* = 272 nm, dimer: *λ_abs_* = 308 nm, trimer: *λ_abs_* = 322 nm, **59**: *λ_abs_* = 348 nm), indicating the extension of π‐conjugation along the polymer chain.

The emission behavior resulting from the alternating BN pattern, however, differs fundamentally from the previous examples: the PPV‐analogue **59** exhibits intense green‐blue fluorescence in solution (*λ_em_* = 379, 494 nm)—hypsochromically shifted compared to the monomeric (*λ_em_* = 574 nm), dimeric (*λ_em_* = 547 nm) and trimeric (*λ_em_* = 528 nm) models—with a remarkable quantum yield of 58%, which is even approaching the 65% of a PMMA film. A huge Stokes shift by Δ*λ* = 140 nm and the appearance of two distinct emission bands in solution indicate dual emission behavior due to an additional twisted intramolecular charge transfer, which is theoretically confirmed. This is fully in line with the absence of the higher energy band in the solid state, where the required structural reorganization in the excited state cannot occur. Instead, aggregation‐induced emission is demonstrated by substantial hypsochromic shifts (Δ*λ* ∼ 50 nm for **59**) with increasing water content (0% to 90%) in thf solutions. DLS indeed confirmed the formation of aggregates with hydrodynamic radii of 60 nm (dimer), 70 nm (trimer), and 106 nm (polymer **59**).

Different polymers with sulfur‐containing bridges and B═N or B─O moieties were recently reported by Helten et al.^[^ ^295^ ^]^ Only for poly(sulfoxide) **60** with B═N units (Figure 14), a bathochromic shift of the UV/vis absorption (*λ_abs_* = 315 nm) compared to corresponding monomers (Δ*λ* = 20–40 nm) was observed. Acids and bases induced selective degradation of these polymers, which holds considerable promise for application as drug delivery agents. The most recent contribution to the field is the heavier B═P congener **61** by the Helten group (*λ_abs_* = 490 nm, *λ_em_* = 595 nm).^[^ ^296^ ^]^ The lower electronegativity of phosphorus causes an even stronger red‐shifted absorption with respect to the corresponding monomer (Δ*λ* = 120 nm) than the B═N analogue **59**. The likewise large Stokes shift (Δ*λ* ∼ 110 nm) was in this case indeed attributed to pyramidalization of the phosphorus centers in the excited state by TD‐DFT calculations on a monomeric model. The poly(phosphaborene) is air‐ and moisture stable in the solid state and exhibited phosphorescence below 100 K.

### 3.2 Group 14: Toward Poly(tetrelene)s and Poly(ditetrelene)s

While there have been several reports of bridged bis(tetrelene)s and bis(ditetrelene)s with element‐carbon and element‐element multiple bonds, respectively,^[^ ^246^, ^297^, ^298^, ^299^, ^300^, ^301^, ^302^, ^303^, ^304^, ^305^, ^306^ ^]^ the development of synthetic protocols for higher oligomers and polymers still represents a major challenge. Due to the inherent lability of the heavier double bonds, they are prone to side reactions and typically require careful handling under an inert atmosphere. Furthermore, decreasing solubility with increasing chain length prevented further chain growth as well as full characterization of presumably polymeric compounds in some cases.

**Scheme 16:**
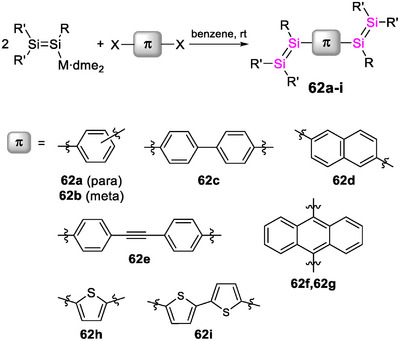
Synthesis of bis(disilene)s **62a–i** (X = Br or I, **62a‐62f**: R = R′ = Tip = 2,4,6‐triisopropylphenyl, M = Li, **62g‐62i**: R = Mes = 2,4,6‐trimethylphenyl, R′ = 1,1,4,4‐tetrakis(trimethylsilyl)‐1,4‐butyl, M = K).

Bis(disilenes) **62a–f** with variable aryl linkers and sterically encumbered 2,4,6‐triisopropylphenyl (Tip) ligands were synthesized by Scheschkewitz et al. in substitution reactions of a lithium disilenide—an anionic silicon analogue of vinyllithium—with the corresponding dihaloarenes (Scheme 16).^[^ ^246^, ^300^, ^303^ ^]^ The *para*‐phenylene‐substituted derivative **62a** served as precursor in a [2 + 2]‐cycloaddition polymerization to the σ,π‐conjugated phenylene‐bridged poly(1,2‐disilacyclobutene) **29** with a Si─Si motif in the repeat unit (vide supra).^[^ ^245^ ^]^ It is worth noting that the in situ generated silene TMS_2_Si = Ad (Ad = 1‐adamantyl) has also been employed in a polymerization to a poly(disilane) with a ─R_2_Si─SiR_2_─CH_2_─ repeat unit.^[^ ^307^ ^]^ In this study by Apeloig et al., the regioselective reaction was either initiated by thermolysis of the silene, releasing radical species, or by the addition of a radical initiator. A comparison of the ^29^Si NMR shifts with molecular model compounds revealed the constitution of the repeat unit.

The Si═Si units in the bis(disilene)s **62a–f** exhibit relatively high deviations from coplanarity with the arylene substituents (dihedral angles: 25 to 75°). Nonetheless, substantial bathochromic shifts by Δ*λ* = 20–70 nm of the absorption maxima (**62a**, **62c–f**: *λ_abs_* = 460–600 nm) compared to the corresponding monomers were obtained, which were partially attributed to conjugation between the Si═Si bonds across the arylene linkers and partially to intramolecular charge transfer transitions. *Meta*‐substitution at the phenylene spacer (**62b**) resulted in an interruption of the conjugation and hence in a hypsochromic shift of the absorption (*λ_abs_* = 450 nm)^[^ ^300^ ^]^ compared to the corresponding *para*‐phenylene‐bridged tetrasila‐butadiene **62a** (*λ_abs_* = 508 nm).^[^ ^246^ ^]^ The biphenyl‐, bis(*para*‐phenylene)‐acetylene‐, naphthalene‐ and anthracene‐bridged **62c‐f** derivatives show orange to NIR fluorescence in solution and in the solid state (*λ_em_* = 570–820 nm).^[^ ^303^ ^]^ No such fluorescence was observed in the case of the thiophene‐, bithiophene‐ and anthracene‐bridged bis(disilene)s **62g–i** with dialkyl‐substitution at the terminal silicon atoms and even larger dihedral angles (80–85°) reported by Iwamoto et al.^[^ ^304^ ^]^

**Figure 15:**
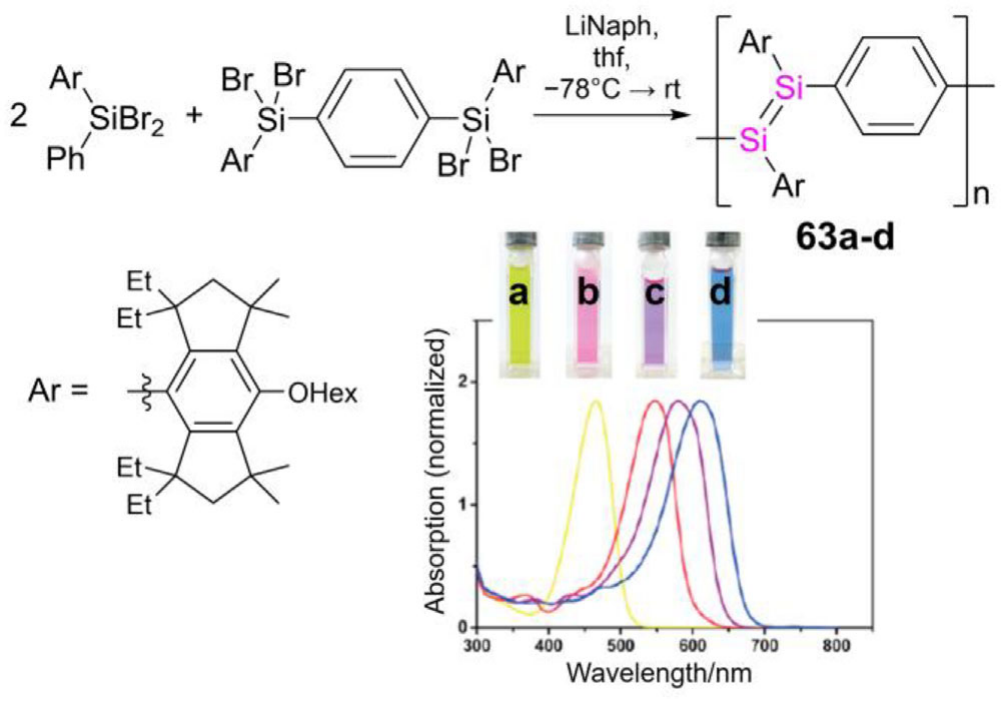
Disilene **63a** (n = 0), bis(disilene) **63b** (n = 1), and oligo(disilene)s **63c,d** (n = 2, 3) with disilenyl end groups are obtained through reductive coupling of a bis(dibromosilane) and a dibromosilane in a 1:2 ratio. Absorption spectra and solutions of **63a‐d** (Naph = naphthalene). Adapted with permission from Ref. [302]. Copyright 2015 American Chemical Society.

In contrast to the *para*‐phenylene‐bridged bis(disilene) **62a**, the analogue by Tamao et al. with *s*‐hydrindacene substituents exhibits coplanarity between the Si═Si units and the central phenyl ring, and even the terminal phenyl groups deviate only slightly with a dihedral angle of 9.0° to the central phenyl group.^[^ ^299^ ^]^ The solubility, however, proved insufficient for the synthesis of longer oligomers, a problem that was overcome by the introduction of hexyloxy groups to the *s‐*hydrindacenyl substituents in **63a–d** (Figure 15).^[^ ^302^ ^]^ In analogy to the insoluble dimer, the synthesis of a mixture of oligo(disilene)s **63a–d** containing up to four Si═Si units was achieved by the reaction of a *para*‐phenylene‐bridged bis(dibromosilane) with a dibromosilane (for end‐capping) in a 1:2 ratio under reductive conditions.

Efficient π‐conjugation along the oligomer chains, resulting from the coplanarity of the phenyl rings and the Si = Si units, was corroborated by remarkable bathochromic shifts of the UV/vis absorption maxima (*λ_abs_* = 470–610 nm) with increasing, albeit limited, chain length (Figure 15): for instance, the tetramer exhibits a red‐shift of Δ*λ* = 145 nm in comparison to the monomer. Furthermore, red fluorescence (*λ_em_* = 610–670 nm) with increasing wavelengths and quantum yields (10%–48%) was observed for the oligomers, in vast contrast to the monomers. The experimentally proposed delocalization of the π‐electrons in the oligo(disilenes) was theoretically confirmed by DFT calculations.^[^ ^246^, ^302^ ^]^ Tamao's disilenes show moderate to good air stability in solution and in the solid state, respectively.^[^ ^299^ ^]^ Consequently and in combination with their luminescent properties, these disilenes constitute promising compounds for the use in optoelectronic devices—in fact, a remarkably stable *s*‐hydrindacene dinaphthyldisilene was applied as the emissive layer in an OLED, albeit with very poor external quantum efficiency (0.014%).^[^ ^308^ ^]^

The *s*‐hydrindacene ligand was further deployed in a coplanar π‐conjugated bis(phosphasilene) with comparable air stability, obtained in a reaction of the bridged disilene and a lithium phosphide.^[^ ^309^ ^]^ Similar to the disilenes, a substantial red shift of the absorption (*λ_abs_* = 449 nm) compared to the monomer (*λ_abs_* = 385 nm) was observed due to the extension of the conjugation. While the absorption of the P═Si dimer is considerably blue‐shifted compared to the Si═Si analogue (*λ_abs_* = 543 nm), the emission maximum (*λ_em_* = 592 nm) differs by only 20 nm. The large Stokes shift in the phosphasilene case was attributed to substantial structural reorganization in the excited state, i.e., twisting about the P─Si bond. As shown recently, the polymerization of arylene‐bridged bis(hydrosilanes) on copper and gold surfaces results in the formation of a sub‐monolayer of poly(disilene)s coordinated to the metal surface.^[^ ^310^ ^]^

**Scheme 17:**
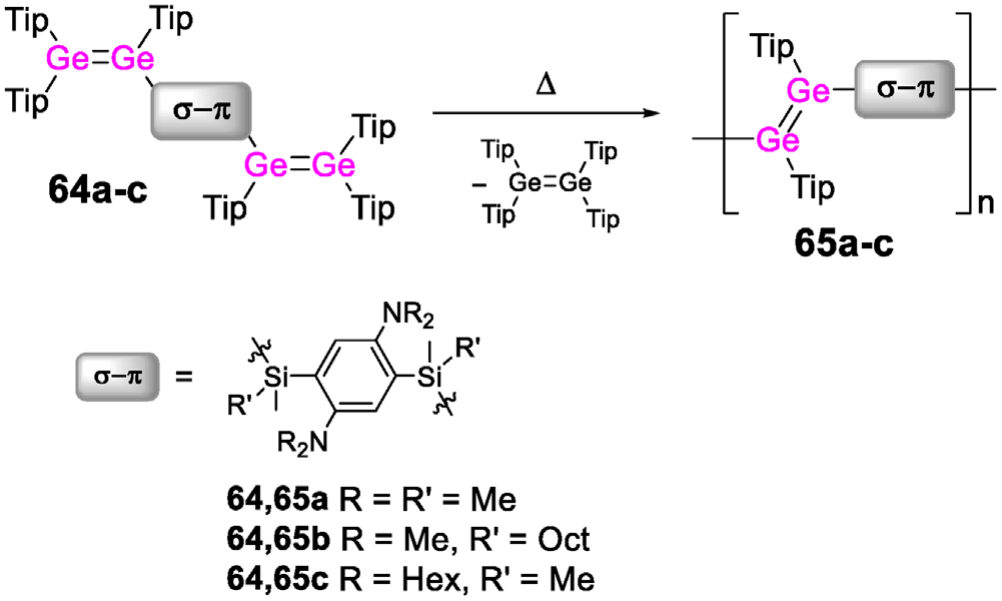
HADMET polymerization of bis(digermene)s **64a–c** provides σ,π‐conjugated poly(digermene)s **65a–c** (Tip = 2,4,6‐triisopropylphenyl).

While analogous germanium compounds are not known as of today, the recently reported synthesis of a silylenephenylene‐bridged bis(digermene) **64a** from a lithium digermenide and the dichlorinated *para*‐disilylarylene linker provided a breakthrough for the development of polymers with Ge═Ge double bonds: the so‐called *heavier acyclic diene metathesis* (HADMET) polymerization of **64a** resulted in the formation of poly(digermene) **65a** and Tip_2_Ge═GeTip_2_ through thermally induced homolytic cleavage of the Ge═Ge double bonds in the monomer (Scheme 17).^[^ ^305^ ^]^ The exclusive formation of the *E*‐isomer was confirmed by comparison of the NMR data with the corresponding monomeric isomers.

The N‐donor in the arylene‐linker was deliberately included in *ortho*‐position to the silyl substituent providing the required intramolecular stabilization of the presumed transient bis(germylene) to avoid undesired side‐reactions. The resulting polymer **65a** precipitates as a yellow solid from the reaction mixture but turns out to be insoluble in all common organic solvents. Therefore, the degree of polymerization was determined indirectly by the amount of the released condensation by‐product (*X_n_* = 23), by a rough estimation based on the particle sizes obtained in DLS measurements (*X_n_* = 26 to 31) and by ^13^C CP/MAS NMR end group analysis (*X_n_* = 23). Further analyses, in particular regarding the photophysical and film‐forming properties and solution NMR spectroscopy, were prohibited by the poor solubility.

The introduction of solubility‐increasing long alkyl chains in the linking units between the Ge═Ge double bonds in bis(digermene)s **64b,c** provided soluble poly(digermene)s **65b,c** with near‐infinite polymer chains,^[^ ^311^ ^]^ which were isolated by precipitation with acetonitrile. On the basis of DLS measurements and diffusion ordered NMR spectroscopy (DOSY), remarkably high degrees of polymerization of *X_n_* = 2100 (**65b**) and 1100 (**65c**) were estimated, supported by the absence of end group signals in the NMR spectra and in line with Carothers’ correlation for the conversion of bifunctional monomers in step‐growth polymerizations. The higher degrees of polymerization in comparison to the insoluble derivative **65a** resulted in relatively high thermal stabilities of **65b,c**: mass loss in the TGA is only evident between 240 and 250 °C, whereas decomposition of **65a** is already observed at 120 °C. DSC and NMR experiments in the case of **65b,c** revealed, however, that degradation is already initiated at ∼170 °C, prior to the actual mass loss.

The high solubility of **65b,c** in different organic solvents enabled the deposition as thin films from polymer solutions. Atomic force microscopy (AFM) and transmission electron microscopy (TEM) revealed the formation of well‐ordered supramolecular cylindrical structures with a lamellar substructure. This self‐organization was attributed to dispersion interactions between the alkyl chains of the linking units as suggested by the single‐crystal X‐ray data of the bis(digermene) monomers and confirmed by powder X‐ray diffractometry of the bulk solids as well as DLS and small angle scattering (SAXS) measurements of solutions. Comparisons of the UV/vis absorption bands (*λ_abs_* = 423, 418 nm) and the NMR data with a monomeric model compound exhibited characteristic bathochromic shifts, indicating the presence of σ,π‐conjugation along the polymer chains, which is also supported by DFT calculations. In a recently developed alternative route toward poly(digermene)s, the reactive Ge(II) centers in a bis(germylene) monomer are stabilized intermolecularly and polymerization was prompted by abstraction of the coordinating N‐heterocyclic carbene donors.^[^ ^306^ ^]^ In principle, this procedure renders the intramolecular stabilization crucial for the HADMET process obsolete and thereby paves the way for variable substitution patterns, for instance without amino groups in the backbone, which are known to quench fluorescence.^[^ ^312^ ^]^ Furthermore, the Me_2_Si bridges might be replaced by Group 13 or Group 15 functional groups or removed altogether to allow for phenylene‐bridging of the Ge═Ge bonds and thus fully π‐conjugated derivatives.

### 3.3 Group 15: Polymers with P═C and P═P Units in the Conjugation Path

**Scheme 18:**
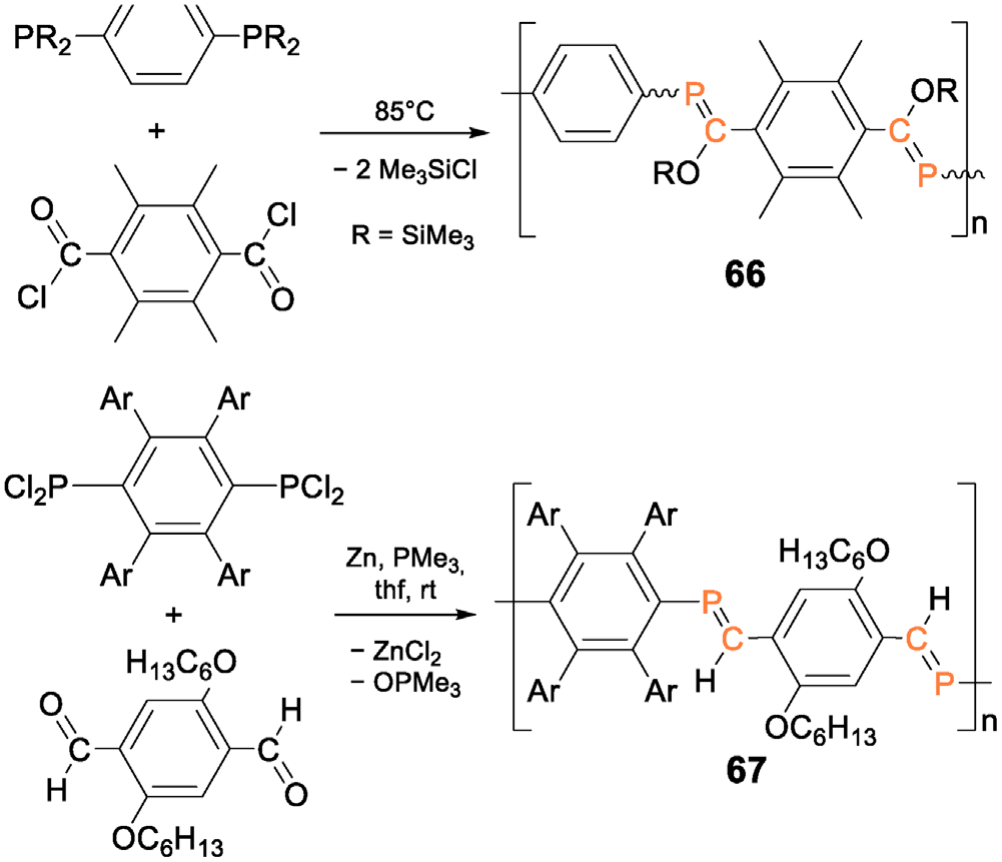
Top: Reaction of a 1:1 mixture of a phenylene‐bridged bis(disilylphosphane) and tetramethylated terephthaloyl chloride to poly(phosphaalkene) **66**. Bottom: The phospha‐Wittig reaction of an arylene‐bridged bis(dichlorophosphane) and a dialdehyde to *E*‐poly(phosphaalkene) **67** (Ar = 4‐*tert*‐butylphenyl).

The first example of a hybrid polymer with an implemented heavier main group multiple bond was the poly(phosphaalkene) **66** reported by Gates et al. in 2002 (Scheme 18).^[^ ^313^ ^]^ It was obtained in a substitution reaction of a *para*‐phenylene‐bridged bis(disilylphophane) and tetramethylated terephthaloyl chloride, followed by [1,3]‐sigmatropic Brook‐type rearrangement to form the P═C double bonds. In the solvent‐free process, the *para*‐arylene‐bridged starting materials were melted together as a neat 1:1 mixture for 21 to 34 h. Subsequent precipitation from a thf solution by addition of hexane at low temperature provided **66** as a yellow solid. ^31^P and ^29^Si NMR spectra revealed the formation of a *Z/E* isomeric mixture (Z/E ∼ 1.1) and ^31^P NMR end group analysis exhibited degrees of polymerization of *X_n_* = 5 to 21.

According to thermogravimetric analysis, the polymer showed reasonable thermal stability up until 190 °C. A slight bathochromic shift of the UV/vis absorption of Δ*λ* ∼ 15–30 nm compared to monomeric and dimeric model compounds supports the presence of π‐conjugation in poly(phosphaalkene) **66**. Exclusive formation of the *Z,Z*‐form, in which π‐conjugation is presumably favored due to the *trans*‐conformation of the arylene groups, is achieved by reversing the steric demand of the linking units of the two components: a durylene‐bridged bis(phosphane) and unsubstituted terephthaloyl chloride instead of the above mentioned starting materials.^[^ ^314^ ^]^ Limited solubility of the obtained product prevented further analyses and a determination of the degree of polymerization in this case. Nonetheless, the soluble part of the pure *Z*,*Z*‐isomer gives rise to a bathochromically shifted absorption at *λ_abs_* = 394 nm compared to the isomeric mixture (*λ_abs_* = 330–340 nm), corroborating the assumed enhanced π‐conjugation.

Protasiewicz et al. employed a *para*‐*tert*‐butylphenyl‐substituted phenylene linker in a phospha‐Wittig reaction with a range of π‐bridged dialdehydes to form poly(phosphaalkene)s.^[^ ^315^ ^]^ The insolubility of the initially obtained derivatives was addressed by employing a phenylene linker with solubility‐enhancing hexyloxy groups in *E*‐poly(phosphaalkene) **67** (Scheme 18). The degree of polymerization amounted, nonetheless, to only *X_n_* = 6 according to end group analysis. The absorption (*λ_abs_* = 445 nm) is identical to that of the corresponding dimer, but the P═C groups provoke a bathochromic shift of Δ*λ* = 20 nm compared to *E*‐poly(phenylenevinylene).

**Scheme 19:**
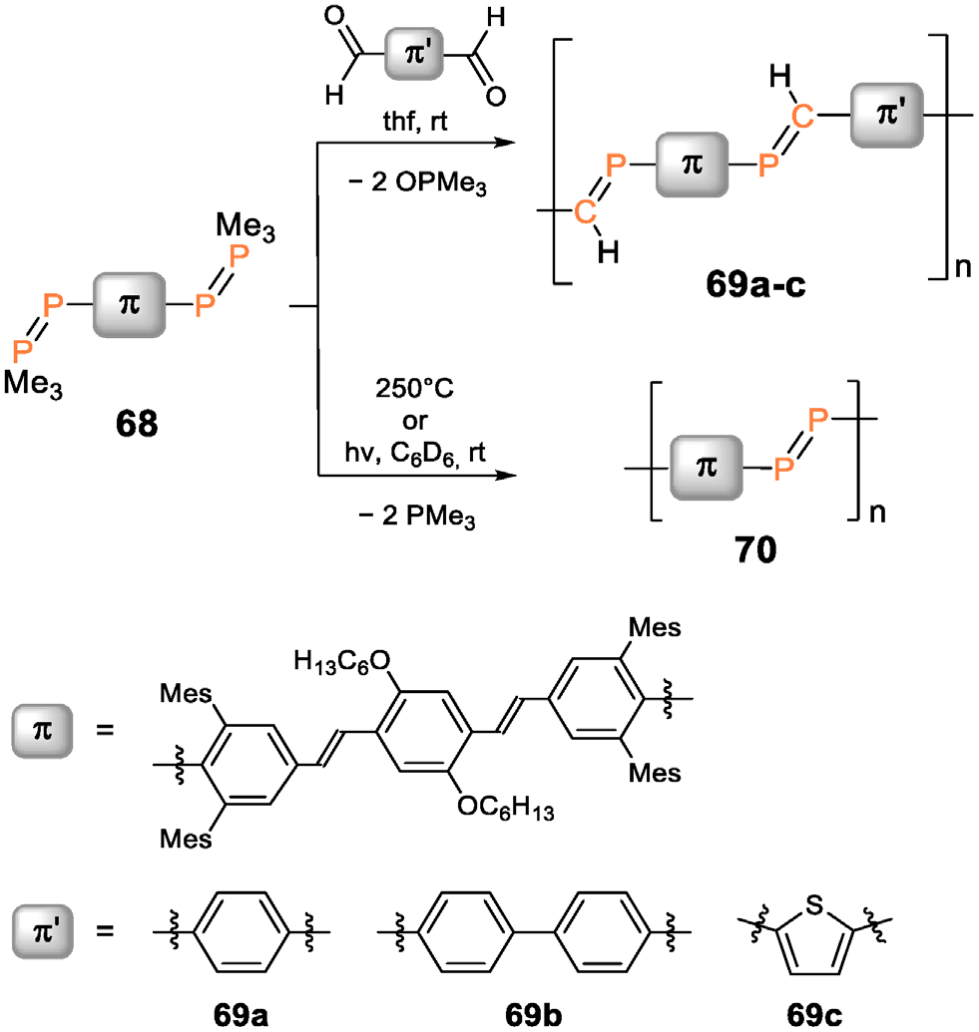
Phospha‐Wittig reaction of arylenevinylene‐bridged bis(diphosphene) **68** with dialdehydes yields poly(phosphaalkene)s **69a–c,** and thermolysis or photolysis provides poly(diphosphene) **70** (Mes = 2,4,6‐trimethylphenyl).

In a conceptually related manner, the corresponding phospha‐Wittig reaction proceeding from bis(diphosphene) **68** provides poly(phosphaalkene)s **69a–c** (Scheme 19) with phenylene, biphenylene, or thiophene linking units in combination with an oligo(phenylenevinylene) linker between the P═C bonds and a degree of polymerization of *X_n_* = 4.5 to 6.5.^[^ ^316^ ^]^

The same arylenevinylene bridged bis(ylide) was used to prepare a poly(diphosphene) **70** with P═P double bonds and *X_n_* = 5.8 via photolytic or thermolytic cleavage of PMe_3_ and formal oligomerization of the bis(phosphinidene). While the absorption of poly(phosphaalkene)s **69a–c** with an extended π‐system as the linking unit are expectedly bathochromically shifted (*λ_abs_* = 420–450 nm) compared to **66** and derivatives reported by Gates et al. (*λ_abs_* = 330–390 nm, vide supra),^[^ ^313^, ^314^ ^]^ the absorption wavelength is not influenced by the chain length as manifest in the comparison with a bis(phosphaalkene) model compound. Nonetheless, poly(phosphaalkene)s **67** and **69a‐c** exhibit green‐blue fluorescence (*λ_em_* = 480–550 nm), which is enhanced in comparison to the model compound, and hence indeed confirms the presence of π‐conjugation. Poly(diphosphene) **70** exhibits the π → π* transition in the same region of the absorption spectrum at *λ_abs_* = 440 nm and an additional n → π* absorption band at a longer wavelength (*λ_abs_* = 481 nm).

## 4 Conclusions and Outlook

We have outlined the impact of different inorganic heteroelement motifs on the conjugation and the resulting photophysical and charge transport properties in hybrid polymers with organic π‐conjugated scaffolds with regard to applications in organic electronics (Table 1). Major progress has been made in this field during the past two decades, which resulted in a large variety of representatives with single heteroatoms in the main chain and in the first examples with diheteroatomic multiple bonds.

**Table 1:** Summary of absorption and emission maxima ranges of selected inorganic‐organic hybrid polymers with p‐block elements and associated proof‐of‐principle applications in polymer electronics.

Heteroelement (motif)	Interaction with conjugation path	*λ*_abs_ (nm)^b)^	*λ*_em_ (nm)^b)^	Application in polymer electronics (selected characteristic device efficiencies)^c)^
B	Direct	350–490	360–650 (690)^d)^	OSC (PCE: up to 2.83%)
	Indirect	450–500	500–610^d)^	–
Ga^a)^	Direct	280–330	380–390	–
	Indirect	330–480	380–640	–
Ge	Direct	270–540	350–490	OLED (EQE up to 24%)
	Indirect	380–770 (830)	420–700 (710)	OSC (PCE up to 6.5%; hole mobilities up to 2.5·10^−2^ cm^2^ V^−1^ s^−1^) OFET (hole mobilities up to 4·10^−2^ cm^2^ V^−1^ S^−1^)
Si–Si/Ge–Ge	–	240–500	350–620	–
Sn	Direct^a)^	–	460–540	–
	Indirect	280–560	540–720	–
P	Direct (P^III^)	280–290	–^d)^	–
	Direct (P^V^)	290–800	330–420 (560)	–
	Indirect (P^III^)	380–520	590^a)^	OLED
	Indirect (P^V^)	400–680	550–560	OLED, OSC (PCE up to 7.0%)
As	Direct^a)^	340	400–490	–
	Indirect	390–680	460–890^d)^	OFET (hole mobility: 0.08 cm^2^ V^−1^ s^−1^) OSC (PCE up to 2.9%)
Bi^a)^	–	310	440	–
B═N	–	290–390	380–600	OFET (hole mobilities up to 0.38 cm^2^ V^−1^ s^−1^)
B═P^a)^	–	490	600	–
Si═Si	–	450–610	570–820	OLED (EQE = 0.014%)
P═Si^a)^	–	450	590	–
Ge═Ge^a)^	–	420	–	–
P═C	–	330–450	480–550	–
P═P^a)^	–	440–480	–	–

^a)^ Only a few derivatives known or explicitly discussed.

^b)^ Upper limit due to bathochromically shifted thin film absorption and emission is provided in parentheses, if discussed.

^c)^ PCE = power conversion efficiency, EQE = external quantum efficiency.

^d)^ Switchable fluorescence observed upon coordination of electrophiles or nucleophiles.

For Group 13, we described recent advances in the chemistry of the well‐established poly(borane)s and the few known examples with gallium. The empty p‐orbital at the heteroelement allows for p,π‐interaction with the π‐framework, which can be interrupted by Lewis base coordination, hinting at applications as sensing materials but also enabling intense charge transfer transitions in notable contrast to the corresponding monomers. The effect of base coordination can be fine‐tuned by the connectivity of the heteroatom.

Similarly, the properties of Group 14 poly(heterole)s strongly depend on the connectivity as well as on the choice of substituents at the heteroelements and in the backbone. *Direct* incorporation of one or two saturated Group 14 centers into the π‐conjugation path gives rise to σ,π‐conjugated hybrid polymers. Although the electron transfer across σ*‐orbitals is by far less efficient, such polymers have been employed in prototypical OLED devices with excellent external quantum efficiencies.

The heteroatom centers in poly(pnictane)s allow for both n,π‐ and σ,π‐interaction. In addition, the lone pairs can be addressed in selective post‐functionalization reactions, such as the reversible coordination of Lewis acids, resulting in unique switchable fluorescence well‐suited for sensing applications. In poly(phosphole)s and derivatives, the heteroelement is not *directly* situated in the conjugation path, but even smaller band gaps are achieved through *indirect* interaction. Poly(phosphole)s have thus been employed in (opto‐)electronic devices with adjustable properties by variation of the oxidation state of phosphorus. Arsenic‐based examples turn out to be much less sensitive toward oxidation than their phosphorus counterparts, which, on the one hand, provides improved air and moisture stability, but on the other hand requires the adjustment of absorption and fluorescence properties by the use of different linking units between the As(III) centers.

While the incorporation of saturated p‐block heteroelements has reached an impressive maturity since the turn of the millennium, the number of compounds with multiple bond motifs in conjugated organic frameworks is considerably lower and applications in devices are still rare. In Group 13, the only known representatives are poly(iminoborane)s with zwitterionic B═N units, which have gained increasing attention in the last decade, and the recently reported first phosphaborene analogue with B═P units. New synthetic routes provided first derivatives with donor‐acceptor character, which in some cases exhibit huge Stokes shifts and dual emission behavior due to twisted intramolecular charge transfer. A development in this regard could be the deliberate increase of the multiple bond character in the B═N units and hence promotion of charge transfer along the polymer chain by the implementation of acceptor units with higher electrophilicity at the boron termini. This should contribute to a deeper understanding of the photophysical properties of poly(iminoborane)s and hence support the development of corresponding purpose‐built materials. Recent advances in the chemistry of low‐coordinate heavier Group 14 compounds have resulted in polymers with Ge═Ge double bonds, which exhibit σ,π‐conjugation across silylene‐phenylene bridging units. Aggregation resulted in ordered structures with lamellar patterns in thin films, as typically required for charge transfer in materials for electronics. Notably, no fluorescence was observed in the poly(digermene)s, in contrast to previously reported oligomers with Si═Si bonds, which exhibit a distinct dependence of the absorption and emission on the chain length. The recent discovery of an alternative synthetic route could provide access to derivatives with variable backbones and different conjugation mechanisms between the Ge═Ge units and the organic π‐system to allow for fine‐tuning of the electronic structure and avoid undesirable quenching of luminescence. Similarly, the stereoactive lone‐pairs in the hitherto only example of a poly(diphosphene) with P═P bonds prohibit fluorescence as well, as manifest in increasing quantum yields in poly(phosphaalkene)s with P═C bonds with decreasing phosphorus content. Derivatization reactions inspired by the rich chemistry of poly(phosphole)s may help to address this issue. Sterically demanding and electronically stabilizing substituents might promote air‐stable derivatives of the typically labile heavier p‐block double bonds, even though air‐ and moisture stability is an inherent issue in the modus operandi of organic electronics and therefore generally circumvented by encapsulation.^[^ ^6^, ^317^, ^318^ ^]^

Recent years have seen an increasing trend toward the incorporation of heavier p‐block elements in hybrid polymers. The toolbox for the construction of such systems is progressively enriched so that corresponding unprecedented combinations can be tackled in the imminent future: for instance, indium, thallium, lead, and antimony‐containing polymers are completely unknown, and in the case of bismuth, only a single example, exhibiting green‐blue fluorescence, has been reported as of yet.

In addition, the huge potential of unconventional multiple bonds between p‐block elements has barely been tapped. For example, diborene units as well as the heavier congeners,^[^ ^319^, ^320^, ^321^, ^322^, ^323^, ^324^, ^325^, ^326^, ^327^, ^328^, ^329^, ^330^, ^331^, ^332^, ^333^, ^334^, ^335^, ^336^ ^]^ although preparatively demanding, may offer rich opportunities for post‐functionalization. Furthermore, copolymerization strategies for the development of donor‐acceptor type polymers are remarkably underrepresented in this field, despite the tendency toward homolytic dissociation^[^ ^104^, ^333^, ^336^, ^337^, ^338^, ^339^ ^]^ and resulting recent advances in the metathetic exchange at heavier multiple bonds.^[^ ^305^, ^340^, ^341^ ^]^

Independent of the employed heteroelement, the nature of the bridging units between the inorganic motifs has been shown to play a crucial role in the adjustment of the photophysical and charge transport properties. A subtle balance between a sufficiently small band gap and matching HOMO and LUMO levels has to be met. Furthermore, the electronic and geometrical constraints introduced through the heteroatom motif need to be reconciled with the organic environment to allow for effective interaction. The impact of structural changes is, for instance, manifest in frequently observed aggregation‐induced absorption and emission in thin films in comparison to corresponding solutions.

With the survey of the structure‐function relationship of various inorganic‐organic hybrid polymers provided herein, further developments toward a targeted fine‐tuning of the electronic and structural features by the incorporation of different (heavier) heteroelements may be facilitated and thus allow to exploit the potential of a modular approach. Interdisciplinary approaches are needed to take full advantage of the exciting developments at the cutting edge of research endeavors until recently dubbed purely academic. We conclude with expressing the hope that this review will contribute to reducing existing barriers between the involved disciplines.

## Conflict of Interests

The authors declare no conflict of interest.

## Data Availability

Data sharing is not applicable to this article as no new data were created or analyzed in this study.
